# Involvement of a truncated MADS-box transcription factor ZmTMM1 in root nitrate foraging

**DOI:** 10.1093/jxb/eraa116

**Published:** 2020-03-05

**Authors:** Ying Liu, Zhongtao Jia, Xuelian Li, Zhangkui Wang, Fanjun Chen, Guohua Mi, Brian Forde, Hideki Takahashi, Lixing Yuan

**Affiliations:** 1 Key Lab of Plant–Soil Interaction, MOE, College of Resources and Environmental Sciences, China Agricultural University, Beijing, China; 2 Lancaster Environment Centre, Lancaster University, Lancaster, UK; 3 Department of Biochemistry and Molecular Biology, Michigan State University, East Lansing, MI, USA; 4 Center for Crop Functional Genomics and Molecular Breeding, China Agricultural University, Beijing, China; 5 University of Warwick, UK

**Keywords:** *AGL17-like*, *ANR1*, lateral root development, MADS-box, maize, nitrate signal, nutrient foraging

## Abstract

Plants can develop root systems with distinct anatomical features and morphological plasticity to forage nutrients distributed heterogeneously in soils. Lateral root proliferation is a typical nutrient-foraging response to a local supply of nitrate, which has been investigated across many plant species. However, the underlying mechanism in maize roots remains largely unknown. Here, we report on identification of a maize truncated MIKC-type MADS-box transcription factor (ZmTMM1) lacking K- and C-domains, expressed preferentially in the lateral root branching zone and induced by the localized supply of nitrate. ZmTMM1 belongs to the AGL17-like MADS-box transcription factor family that contains orthologs of ANR1, a key regulator for root nitrate foraging in Arabidopsis. Ectopic overexpression of *ZmTMM1* recovers the defective growth of lateral roots in the Arabidopsis *anr1 agl21* double mutant. The local activation of glucocorticoid receptor fusion proteins for ZmTMM1 and an artificially truncated form of AtANR1 without the K- and C-domains stimulates the lateral root growth of the Arabidopsis *anr1 agl21* mutant, providing evidence that *ZmTMM1* encodes a functional MADS-box that modulates lateral root development. However, no phenotype was observed in *ZmTMM1-RNAi* transgenic maize lines, suggesting a possible genetic redundancy of *ZmTMM1* with other *AGL17-like* genes in maize. A comparative genome analysis further suggests that a nitrate-inducible transcriptional regulation is probably conserved in both truncated and non-truncated forms of ZmTMM1-like MADS-box transcription factors found in grass species.

## Teaser

A MADS-box transcription factor transcriptionally up-regulated and modulating lateral root development in response to local nitrate supply exists as a truncated form unique to grass species.

## Introduction

Natural and agricultural soils present a large spatial and temporal diversity in nutrient distribution (Giehl and von Wirén, 2014). Plant root systems exhibit highly flexible plasticity to explore the fluctuating nutrients in soils (Giehl *et al.*, 2014). Nitrate, as the main inorganic nitrogen source in aerobic soils, is heterogeneously distributed due to its high solubility and rapid mobility in soil solution (Lark *et al.*, 2004; Miller *et al.*, 2007). To capture the unevenly distributed nitrate, plants stimulate lateral root (LR) growth in nitrate-rich patches, which has been reported as a typical nutrient-foraging response in many plant species, including Arabidopsis, barley, rice, and maize (Drew, 1975; Zhang and Forde, 1998; Wang *et al.*, 2002; Remans *et al.*, 2006; Liu *et al.*, 2008; P. Yu *et al.*, 2014). Lines of experimental evidence suggest that nitrate *per se* supplied locally to roots acts as a signaling molecule to modulate LR growth (Zhang and Forde, 1998; Remans *et al.*, 2006; Ho *et al.*, 2009; Bouguyon *et al.*, 2015). In Arabidopsis, local nitrate is sensed by the nitrate transceptor NRT1.1/NPF6.3, which then triggers the signaling pathway involving the MADS-box transcription factor ANR1 to stimulate LR proliferation in a nitrate-enriched zone (Zhang and Forde, 1998; Remans *et al.*, 2006; Bouguyon *et al.*, 2015). In addition, local nitrate can inhibit NRT1.1-facilitated auxin redistribution in LR primordia, allowing auxin accumulation for LR emergence and elongation (Krouk *et al.*, 2010; Mounier *et al.*, 2014).

MADS-box transcription factors govern diverse developmental processes in plants, including floral formation, pollen maturation, fruit development, and root development (Theissen *et al.*, 2000; Ng and Yanofsky, 2001; Smaczniak *et al.*, 2012; Alvarez-Buylla *et al.*, 2019). The MIKC-type, also termed as type II MADS-box transcription factors, contain four conserved domains: the MADS-box (M-) domain, the intervening (I-) domain, the keratin-like (K-) domain, and the C-terminal (C-) domain (Alvarez-Buylla *et al.*, 2000). Many MIKC-type MADS-box genes, particularly *AGL17-like* genes, control root development (Becker and Theissen, 2003; Alvarez-Buylla *et al.*, 2019). In Arabidopsis, three out of four members within the *AGL17-like* clade, specifically *AGL17*, *AGL21*, and *ANR1*, are preferentially expressed in roots (Rounsley *et al.*, 1995; Zhang and Forde, 1998; Alvarez-Buylla *et al.*, 2000; Burgeff *et al.*, 2002; C. Yu *et al.*, 2014). *ANR1* promotes LR elongation under local nitrate supply via enhancing LR meristem activity (Zhang and Forde, 1998; Gan *et al.*, 2012), while *AGL21* is required for sustaining LR growth under N deficiency by increasing local auxin biosynthesis (L.-H. Yu *et al.*, 2014). In rice, four out of five members of the *AGL17-like* genes, *OsMADS23, OsMADS25*, *OsMADS27*, and *OsMADS57*, regulate root development (Puig *et al.*, 2013). *OsMADS25* promotes growth of both the primary root (PR) and the LR, coinciding with the elevated auxin levels in the presence of nitrate, suggesting the roles of this *AGL17-like* gene in root nitrate foraging (C. Yu *et al.*, 2015; Zhang *et al.*, 2018). *OsMADS57* also promotes seminal and adventitious root elongation, and, in parallel, increases root to shoot nitrate translocation by modulating gene expression of NRT2 nitrate transporters (Huang *et al.*, 2019). Interestingly, a monocot-exclusive miRNA, miR444, specifically targets *OsMADS23*, *OsMADS27*, and *OsMADS57* for degradation, and inhibits LR growth in rice (Yan *et al.*, 2014).

Roots are shaped differently in monocot and dicot species, as they develop the overall root architecture composed of multiple root types with distinct anatomic features. The maize root system is composed of multiple root types including the embryonic primary and seminal roots, and the post-embryonic crown roots initiating from below-ground nodes and brace roots from above-ground nodes (Hochholdinger *et al.*, 2004). LR development across the individual root types in maize shows distinct morphological responses to nitrate heterogeneity (P. Yu *et al.*, 2014). Specifically, local nitrate supply mainly promotes LR elongation in the primary, seminal, and crown roots, while both LR initiation and elongation are stimulated in brace roots (P. Yu *et al*., 2014, 2015, 2016). Yet, the molecular mechanisms and key regulators of nitrate-foraging responses remain unclear in maize.

Transcriptome analysis of nitrate-responsive genes implicates that many transcription factors participate in nitrate-foraging responses in maize roots, including several MADS-boxes (Liu *et al.*, 2008; Yu *et al.*, 2016). In this study, we report functional characterization of a novel truncated MIKC-type MADS-box transcription factor gene *ZmTMM1* identified as a local nitrate-responsive gene from our previous transcriptome analysis of maize roots (Liu *et al.*, 2008). Sequence analysis reveals that *ZmTMM1* encodes a previously uncharacterized truncated form of an AGL17-like MADS-box lacking K- and C-domains. Functional analysis of *ZmTMM1* reveals its involvement in root nitrate-foraging responses, complementing the function of Arabidopsis *ANR1* and *AGL21*. Moreover, based on the comparative genome analysis, we uncover the local nitrate-inducible transcriptional regulation of *ZmTMM1* as a common feature conserved within orthologs present in grass species.

## Materials and methods

### Plant material and growth conditions

Maize (*Zea mays* L.) inbred line B73 was used for gene cloning, gene expression, and root morphological analyses. Maize genotype Hi II was used to generate *ZmTMM1-RNAi* transgenic lines. *Arabidopsis thaliana* accession Columbia (Col-0) served as the wild type. The *anr1* and *agl21* knockout mutants were *dSpm* transposon insertion lines identified from the Sainsbury Laboratory *Arabidopsis thaliana* collection (SLAT). Homozygotes of *anr1* and *agl21* were screened from SLAT mutant pool N40108 by PCR (Gan *et al.*, 2005). Specifically, for the *anr1* homozygous mutant, a gene-specific primer SG2 and a *dSpm* primer dSpm1 were used to verify the transposon insertion, while gene-specific primers SG2 and SG10 were used to confirm the homozygosity of the insertion (Gan *et al.*, 2005). For the *agl21* homozygous mutant, a gene-specific primer SG4 and a *dSpm* primer dSpm8 were used to verify the transposon insertion, and gene-specific primers SG3 and SG6 were used to confirm the homozygosity of the insertion. These primers are listed in Supplementary Table S2 at *JXB* online. The double mutant *anr1 agl21* (*dko* mutant) was generated by crossing the *anr1* and *agl21* single mutants. Rice (*Oryza sativa* L. *japonica*) genotype Nipponbare was used for gene expression analysis.

#### Maize culture

For hydroponic experiments under uniform nutrient supply, maize seeds were surface sterilized in 10% (v/v) H_2_O_2_ for 30 min, and germinated in paper rolls (Gu *et al.*, 2013). Six days after germination, endosperms were removed and seedlings with two visible leaves were transferred to Hoagland solution (pH 5.7) containing 2 mM NH_4_NO_3_ as the N source. Nutrient solution was renewed every 2 d. For N starvation, NH_4_NO_3_ was removed from the Hoagland solution. For nitrate or ammonium resupply, 4 mM KNO_3_ or 2 mM (NH_4_)_2_SO_4_ was added, respectively. All seedlings were grown in a climatic chamber under 14 h/10 h (light/dark) and 28 °C/22 °C rhythms at a light intensity of 300 µmol m^–2^ s^–1^ and 60% humidity. For split-root experiments, a two-compartment split-root hydroponic system developed previously (Liu *et al.*, 2008) was used as described below. Maize seedlings were pre-cultured in the Hoagland solution with 0.5 mM KNO_3_ for 9 d, and then the primary and seminal roots were removed so that four crown roots remained on the seedlings. After 3 d of N starvation, these four crown roots were equally divided into two groups and transferred to a two-compartment split-root system containing 1 mM KNO_3_ or 0.5 mM (NH_4_)_2_SO_4_ in the +N compartment and 0.5 mM K_2_SO_4_ in the –N compartment, respectively.

#### Arabidopsis culture

Arabidopsis seeds were surface sterilized in 70% ethanol with 0.05% Triton X-100. For Arabidopsis phenotyping experiments, N-free half-strength Murashige and Skoog (MS; Murashige and Skoog, 1962) mineral salts medium supplied with 1% sucrose, 2.5 mM MES (pH 5.8), and 1% Difco agar (BD Biosciences) was used as basic medium, and the N sources were added to be adjusted to final concentrations as indicated in the figures. Seedlings were cultured vertically in a growth chamber under 16 h/8 h (light/dark) diurnal cycles with temperatures during the day and night controlled at 22 °C and 20 °C and with a light intensity of 120 µmol m^–2^ s^–1^. In experiments with homogeneous N supply, seedlings were cultivated on N-free half-strength MS medium supplemented with 1 mM KNO_3_ as the N source for 12 d. For Arabidopsis split-root experiments, seedlings were pre-cultured on N-free half-strength MS medium supplemented with 1 mM KNO_3_ for 6 d, and then PRs were excised, leaving only two first-order LRs. After a recovery for 3 d, plants with two comparable LRs were transferred to the vertical split-agar plates containing 1 mM KNO_3_ in the +N side and 0.5 mM K_2_SO_4_ in the –N side. Root phenotypes were analyzed at 6 d after the transfer. For the local activation experiments using the glucocorticoid receptor (GR) fusion system, PRs of 9-day-old seedlings were pruned to have two LRs, and these LRs were transferred onto the vertically segmented agar plates containing 1 µM dexamethazone (DEX) in the +DEX side and 0.01% ethanol as a mock control in the –DEX side. Segmented agar plates for the local DEX treatment were supplied with 1 mM KNO_3_ or 0.5 mM Gln as the N source. Root phenotypes were analyzed at 8 d after the transfer.

#### Rice culture

For split-root experiments, rice seedlings were pre-cultured in IRRI rice culture solution (Yoshida *et al.*, 1976) containing 1 mM NH_4_NO_3_ as the N source for 25 d. After 3 d of N starvation, rice plants were transferred into the two-compartment split-root system (Liu *et al.*, 2008) containing 1 mM KNO_3_ in the +N compartment and 0.5 mM K_2_SO_4_ in the –N compartment. Roots were harvested for gene expression analysis at 12 h after the transfer to validate the effect of the localized nitrate treatment.

### Root growth measurements

Arabidopsis and maize roots were scanned by an Epson V700 scanner at a resolution of 300 dpi. Root lengths were measured by Image J or WinRHIZO Pro 2007 software. The lengths of the axis root and LR branching zone were measured and the visible LR root number was counted. To assay LR initiation and primordial development, Arabidopsis roots were cleared by HCG solution (chloral hydrate:water:glycerol=8:3:1) for 10 min, and LR primordia embedded in PR were counted under differential interference contrast (DIC) microscopy. Developmental stages of LR initials were classified according to Malamy and Benfey (1997).

### Statistical analysis

All statistical analyses were performed by SigmaPlot 11.0 software. A paired *t*-test or one-way ANOVA was used to test the statistical significance, and the *P*-value of each statistical anaylsis is described in the figure legends.

### *In situ* RNA hybridization

*In situ* RNA hybridization was performed as previously described (Gu *et al.*, 2013). Maize roots were harvested from the hydroponic system and fixed in FAA solution (formaldehyde, ethanol, acetic acid=10%, 50%, 5%) overnight at 4 °C. Thin sections of paraffin-embedded roots of 8–12 μm thickness were generated for the hybridization. A 115 bp gene-specific region of the *ZmTMM1* coding sequence, which was confirmed by quantitative real-time PCR (qRT-PCR), was selected for synthesis of digoxigenin (DIG)-labeled RNA sense and antisense probes *in vitro*. The hybridization reaction was conducted for 24 h at 50 °C. Anti-DIG alkaline phosphatase-conjugated antibody and the NBT (nitro-blue tetrazolium)/BCIP (5-bromo-4-chloro-3-indolyl phosphate) staining method were used to visualize the tissue-specific localization of *ZmTMM1* transcript expression in root sections. Photographs were taken by Olympus BX51 microscope (Japan).

### qRT-PCR

Total RNA was extracted from 50–100 mg of frozen root samples using TRIzol (Invitrogen) solution following the manufacturer’s protocol. Genomic DNA was removed by RQ1 RNase-Free DNase (Promega). Template cDNA was synthesized from 1 µg of total RNA using M-MLV reverse transcriptase (Promega) and oligodT(18) primers. qPCR was conducted by the ABI 7500 Real-Time PCR System (Applied Biosystems) using SYBR Green real-time PCR Master Mix Kit (TOYOBO). Gene expression levels were normalized relative to *ZmGAPDH* and *ZmTUB4* in maize, *AtUBQ10* in Arabidopsis, and *OsACTIN* in rice as internal controls, and by using the ΔΔCt method (Pfaffl, 2001). Gene-specific primers are listed in Supplementary Table S2.

### Generation of transgenic plants

Transgenic Arabidopsis lines (*dko-p35S::ZmTMM1*) were generated by introducing a construct to express *ZmTMM1* under the control of the *Cauliflower mosaic virus* (CaMV) *35S* promoter in the background of the *dko* mutant. The coding sequence of *ZmTMM1* was cloned into the pMD19-T (TaKaRa) vector, and then subcloned into a plant transformation vector pPT-Hyg (Yuan *et al.*, 2007). ZmTMM1–GR, ANR1S–GR, and ANR1–GR constructs were produced by fusing GR protein to the C-terminus of ZmTMM1, ANR1S, and ANR1 protein-coding regions, and inserting these GR fusions downstream of the CaMV *35S* promoter. The GR fusion constructs were subsequently introduced into the *dko* mutant. The promoter–green fluorescent protein (GFP)–nuclear localization sequence (NLS) constructs for *ANR1* and *AGL21* were made by cloning PCR-amplified 2 kb promoter regions into pENTR/D-TOPO (Invitrogen) and integrating the inserts into a Gateway compatible binary vector pBGGN (Funakoshi, Japan), which allows *in situ* analysis of promoter activities based on expression of GFP fused with an NLS. Arabidopsis was transformed by the floral dipping method facilitated by *Agrobacterium tumefaciens* GV3101 (Clough and Bent, 1998). Transgenic plants made by using pPT-Hyg and pBGGN vectors were selected by hygromycin and basta resistance, respectively, and homozygous T_3_ lines were used for subsequent experiments. For studying ZmTMM1 subcellular localization, GFP was fused to the C-terminus of the ZmTMM1 protein-coding region and inserted into the pBI121-GFP vector to be expressed under the control of the CaMV *35S* promoter. The *p35S::ZmTMM1-GFP* fusion construct was transiently expressed in onion epidermal cells. To generate the *ZmTMM1-RNAi* construct, a 115 bp coding sequence of *ZmTMM1* was inserted into an RNAi vector pTCK303 to be expressed under the control of the maize *Ubiquitin 1* gene promoter (Wang *et al.*, 2004). The *pUBQ::ZmTMM1-RNAi* vector was introduced into *A. tumefaciens* EHA105 to transform maize genotype Hi II. Three independent *ZmANR1-RNAi* transgenic maize lines were selected by phosphinothricin (PPT) resistance and further backcrossed with wild-type B73 twice.

### Phylogenetic analysis

Protein sequences encoded by the *AGL17-like* genes in *A. thaliana*, *Brassica rapa*, *Glycine max*, *O. sativa*, *Brachypodium distachyon*, *Sorghum bicolor*, and *Z. mays* were obtained from the PlantGDB database. The phylogenetic tree was generated by MEGA4 software (https://www.megasoftware.net/mega4/) via the Neighbor–Joining method. Numbers shown at the nodes indicate the bootstrap values expressed as percentages obtained from 1000 trials. Motifs were identified by the MEME algorithm (http://meme-suite.org/tools/meme). Gene structure annotation was conducted by the yrGATE algorithm (http://www.plantgdb.org/prj/yrGATE/).

### Accession numbers

Arabidopsis: *ANR1* (*AT2G14210*), *AGL16* (*AT3G57230*), *AGL17* (*AT2G22630*), and *AGL21* (*AT4G37940*); maize: *ZmTMM1* (*GRMZM2G044408*), *ZmMADS2* (*GRMZM2G316366*, *GRMZM2G492156*), *GRMZM2G055782*, and *GRMZM2G032905*; rice: *OsMADS23* (*LOC_Os08g33488*), *OsMADS25* (*LOC_Os04g23910*), *OsMADS27* (*LOC_Os02g36924*), *OsMADS57* (*LOC_Os02g49840*), and *OsMADS61* (*LOC_Os04g38770*, *LOC_Os04g38780*).

## Results

### Identification of a truncated MIKC-type MADS-box gene *ZmTMM1* in maize

**Fig. 1.**
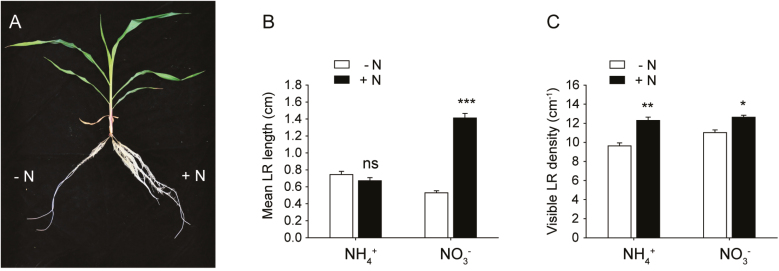
Development of maize LRs in response to localized ammonium or nitrate supply. Maize seedlings with four crown roots were subjected to a two-compartment split-root system which contained either 1 mM KNO_3_ or 0.5 mM (NH_4_)_2_SO_4_ in the +N compartment, and 0.5 mM K_2_SO_4_ in the –N compartment. Root growth was measured at 5 d after transfer. (A) Root growth of maize seedlings in a two-compartment split-root system. (B) Mean LR length. (C) Visible LR density. Data represent means ±SE (*n*=5 replicates; each replicate represents two seedlings). Asterisks indicate significant differences between the roots grown in the +N and –N compartments at *P<0.05; **P<0.01; ***P<0.001; ns, not significant (Student’s *t*-test).

To investigate the effect of local N supply on maize root development, we developed a split-root hydroponic system (Fig. 1A; Liu *et al.*, 2008). After 5 d of local nitrate treatments, the mean length of LRs increased up to 2.5-fold compared with roots growing in the N-free compartment, and the visible LR density increased by 10% (Fig. 1B, C). This indicated that the localized supply of nitrate promoted both LR branching and LR elongation in maize. In contrast, the localized supply of ammonium significantly stimulated LR branching but not LR elongation (Fig. 1B, C).

To uncover the mechanism underlying LR proliferation in response to local nitrate supply, a microarray experiment was conducted in our previous study for identifying nitrate-responsive genes (Liu *et al.*, 2008). A MADS-box transcription factor gene (*GRMZM2G044408*) was identified as a nitrate-responsive gene showing 4-fold up-regulated expression after 1 h of local nitrate treatment. By conducting 5'- and 3'-RACE, we confirmed the full-length transcript of *GRMZM2G044408* that contains a 270 bp ORF, encoding a polypeptide with 89 amino acid residues, which was named truncated MIKC-type MADS-box transcription factor, ZmTMM1. According to the conservation of the DNA-binding domain, *ZmTMM1* was assigned to the *AGL17-like* clade of MADS-box genes with 83% similarity at the protein level to Arabidopsis ANR1 (Zhang and Forde, 1998; Gan *et al.*, 2012).

**Fig. 2.**
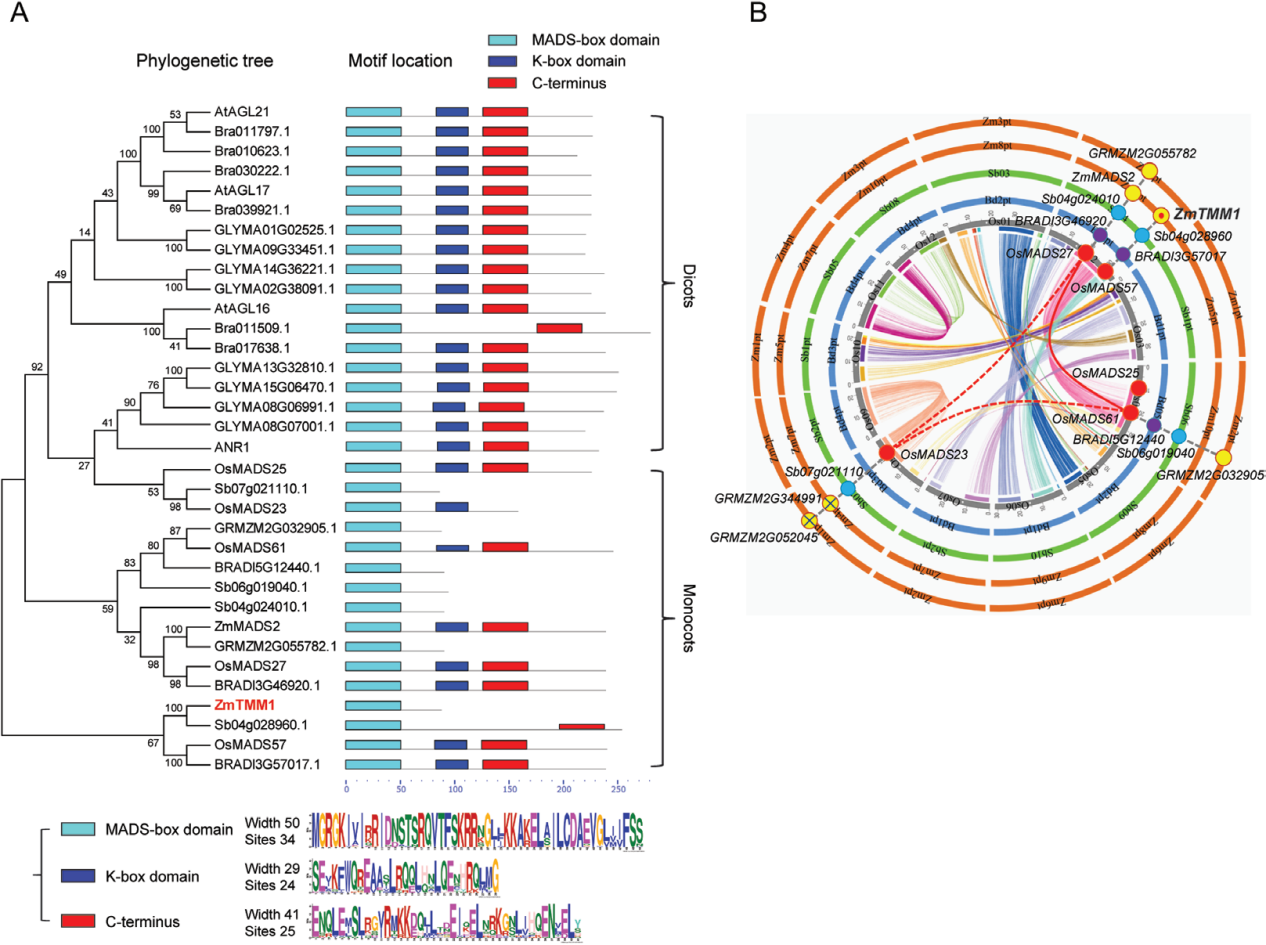
Phylogenetic, motif, and comparative genome analysis of *AGL17-like* genes in plants. (A) Phylogenetic tree and motif analysis of AGL17-like MADS-box transcription factors. Protein sequences encoded by *AGL17-like* genes in *Arabidopsis thaliana*, *Brassica rapa*, *Glycine max*, *Oryza sativa*, *Brachypodium distachyon*, *Sorghum bicolor*, and *Zea mays* were used for the phylogenetic and motif analysis. ZmTMM is highlighted in red. Numbers shown at the nodes indicate the bootstrap values expressed as percentages. (B) Comparative genome analysis of *AGL17-like* genes in grass species. Rings in different colors represent genomes of different species: rice, gray; Brachypodium, blue; sorghum, green; maize, brown. Spots in different colors indicated on the rings represent *AGL17-like* genes in grass species: rice, red; Brachypodium, purple; sorghum, blue; maize, yellow. Red solid line represents intragenomic collinearity in rice. Red dashed lines represent intragenomic micro-collinearity in rice. Gray dashed lines represent interspecific collinearity across the grass species.

Phylogenetic analysis showed that *AGL17-like* genes were subdivided into dicot- and monocot-specific clades (Fig. 2A). Motif analysis revealed that a typical complete MIKC-type MADS-box transcription factor contains four domains: the M-domain, I-domain, K-domain, and C-domain. All *AGL17-like* genes in dicots encoded the complete MIKC-type MADS-box protein structure, whereas the truncated *AGL17-like*-encoded MADS-box proteins existing only in monocots lacked either the K- or C-domain, or even both. Interestingly, ZmTMM1 was devoid of both the K- and C-domains, leaving the remaining portion to be present only with the conserved M-domain and I-domain (Fig. 2A).

Next, we examined how the truncated MADS-box proteins would have been generated in monocot species. An intron exists in the coding sequence of *AGL17-like* MADS-box genes, being inserted into the gap between the I- and K- domains (Parenicová et al., 2003; Supplementary Fig. S1; Supplementary Table S1). *AGL17-like* genes in Arabidopsis contain such introns with a length of 1137–2133 bp, and rice genes, except for *OsMADS25*, with a length of 2755–5184 bp, respectively (Supplementary Fig. S1). Notably, due to the insertion of the introns, *OsMADS61* was incorrectly annotated into two tandem genes, *Os04g38770* and *Os04g38780*, and *ZmMADS2* was mis-annotated into two tandem genes, *GRMZM2G316366* and *GRMZM2G492156* (Supplementary Table S1; Schreiber *et al.*, 2004; Puig *et al.*, 2013). Besides being present and spliced as an intron of a gene encoding the complete MIKC-type MADS-box protein, the existence of such an extremely large size (>9–10 kb) of a gap in a genomic region may cause termination of transcription, producing a truncated MADS-box gene transcript containing only the M- and I-domains, such as *BRADI5G12440* in *Brachypodium*, *Sb07g021110* in sorghum, and *GRMZM2G055782* and *GRMZM2G032905* in maize. The second halves separately annotated downstream in the genome could represent genes encoding the K- and C-domains (Supplementary Table S1). In addition, due to the genome duplication and the chromosome fusion that reconstructed the genome of grass species (Bolot *et al.*, 2009), some MADS-box genes might even have lost these second halves of fragments on the genome, such as *ZmTMM1* in maize, and *Sb04g024010* and *Sb06g019040* in sorghum (Supplementary Table S1). Taken together, the comparative genome analysis provided evidence that *ZmTMM1* encodes a novel truncated *AGL17-like* gene product exclusively identified in grass species.

### *ZmTMM1* is expressed in root xylem parenchyma cells and outer layer cortical cells

**Fig. 3.**
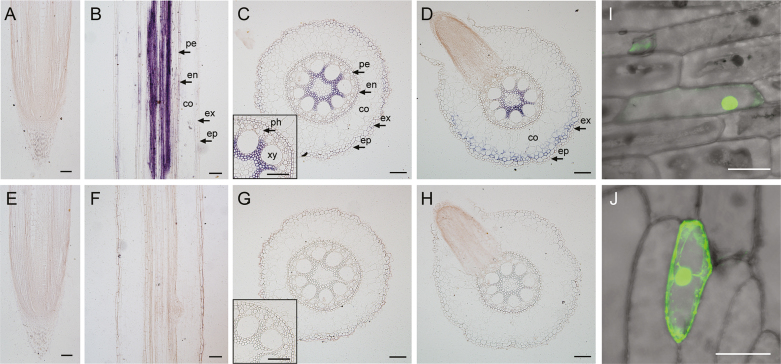
Tissue-specific and subcellular localization of *ZmTMM1*. (A–H) *In situ* RNA hybridization of *ZmTMM1* in maize roots. Maize seedlings were cultivated under 4 mM KNO_3_ for 12 d, and crown roots were harvested. Antisense probes (A–D) and sense probes (E–H) were used to determine the localization of *ZmTMM1* mRNA. Longitudinal sections of crown root tips (A and E); longitudinal sections of crown roots in the maturation zone (B and F); cross-sections of crown roots in the maturation zone (C and G); cross-sections of crown roots with emerging LRs (D and H). ep, epidermis; ex, exodermis; co, cortex; en, endodermis; pe, pericycle; ph, phloem; xy, xylem. Scale bars=100 µm. (I and J) Subcellular localization of ZmTMM1::GFP fusion protein (I) and GFP control (J) in onion epidermal cell. Scale bars=100 µm.

Similar to other *AGL17-like* genes (Burgeff *et al.*, 2002; Becker and Theissen, 2003; Puig *et al*, 2013), *ZmTMM1* was preferentially expressed in maize roots (Supplementary Fig. S2). At the tissue level, *in situ* RNA hybridization showed that *ZmTMM1* transcript was not detected in either crown root tips or LR tips (Fig. 3A, D). However, along the longitudinal section of maize crown roots, *ZmTMM1* transcript was detected in the central cylinder of the root maturation zone (Fig. 3B). The cross-section further revealed that *ZmTMM1* transcript is mainly localized in xylem parenchyma cells of mature roots (Fig. 3C). In the LR branching zone, *ZmTMM1* accumulated in both xylem parenchyma cells and outer layer cortical cells surrounding the emerging LR (Fig. 3D). In contrast, the sense probes for *ZmTMM1* did not generate a detectable signal (Fig. 3E–H). Additionally, by transiently expressing ZmTMM1–GFP fusion protein in onion epidermal cells, ZmTMM1 was shown to localize in the nucleus, which is considered prerequisite for this protein being a transcription factor (Fig. 3I).

### *ZmTMM1* is up-regulated by local nitrate in the LR branching zone

**Fig. 4.**
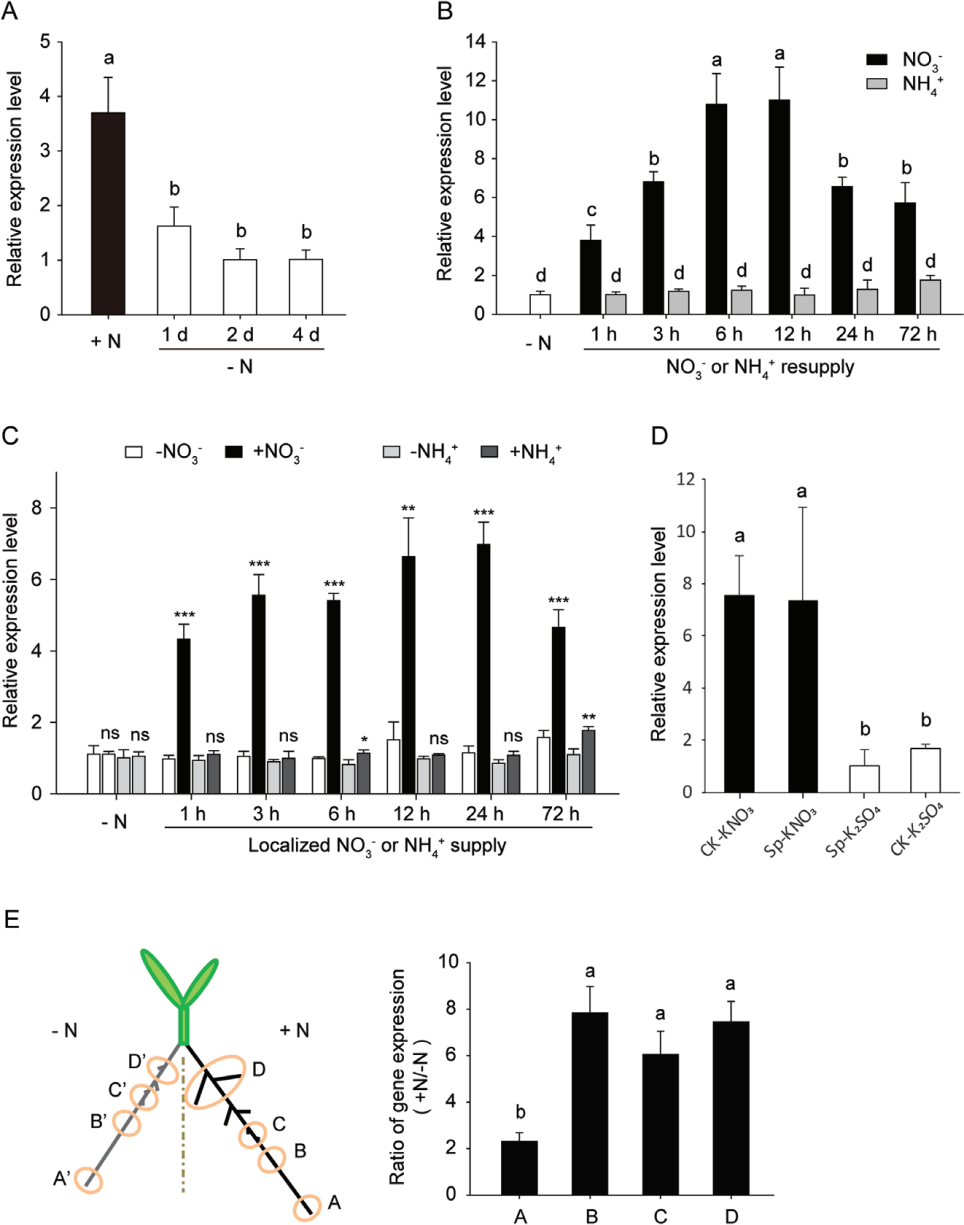
Regulation of *ZmTMM1* in maize roots in response to different N availability. (A) *ZmTMM1* transcript levels changing in response to N deprivation. Maize seedlings were pre-cultured with 2 mM NH_4_NO_3_, and then transferred to N-free solution. (B) Time course analysis of changes in *ZmTMM1* transcript levels in response to the homogenous resupply of nitrate or ammonium. After N starvation for 4 d, maize seedlings were resupplied with either 4 mM KNO_3_ or 2 mM (NH_4_)_2_SO_4_. (C) *ZmTMM1* transcript levels in response to local supply of nitrate or ammonium in a split-root system. Maize seedlings with four crown roots were cultivated in a split-root system supplemented with either 1 mM KNO_3_ or 0.5 mM (NH_4_)_2_SO_4_ in the +N compartment, and 0.5 mM K_2_SO_4_ in the –N compartment. (D) *ZmTMM1* is regulated by local nitrate rather than systemic N signal. CK-KNO_3_, roots in both compartments of the split-root system homogeneously treated with nitrate (1 mM KNO_3_); Sp-KNO_3_, roots in the +N compartment (1 mM KNO_3_) of the split-root system; Sp-K_2_SO_4_, roots in the –N compartment (0.5 mM K_2_SO_4_) of the split-root system; CK-K_2_SO_4_, roots in both compartments of the split-root system subjected to N deprivation (0.5 mM K_2_SO_4_). Relative expression levels of *ZmTMM1* after 8 h of treatment are shown. (E) Ratio of *ZmTMM1* transcript levels between the root segments in the +N and –N compartments of the split-root system. Maize crown roots were harvested from the split-root system after 12 h of treatment and divided into four regions: A, root tip; B, LR initiation region; C, LR elongation region; D, mature LR region. Roots were harvested for gene expression analysis at the indicated time points. The relative transcript level of *ZmTMM1* was determined by qPCR and normalized by maize *Tubulin 4* (*AJ420856*). Data represent means ±SD (*n*=3 replicates; each replicate represents a single seedling). Different letters represent significant differences among means at *P*<0.05 (Tukey’s test). Asterisks indicate significant differences between the roots grown in the +N and –N compartments at *P<0.05; **P<0.01; ***P<0.001; ns, not significant (Student’s *t*-test).

Next, we investigated the response of *ZmTMM1* at the transcriptional level to different N availabilities. First, the expression of *ZmTMM1* was found to be repressed by N starvation (Fig. 4A). In response to N resupply, *ZmTMM1* was specifically up-regulated following addition of nitrate, showing an increase starting after 1 h and reaching maximum levels at 6–12 h by 10-fold in the time course (Fig. 4B). In contrast, the *ZmTMM1* transcript level was unaffected by ammonium resupply. In a split-root system, *ZmTMM1* was specifically up-regulated by the localized nitrate supply rather than ammonium (Fig. 4C), while the root N status and transcript levels of an N-responsive marker gene *ZmGS1.1* (Guo *et al.*, 2015) increased in response to both the localized supply of nitrate and ammonium (Supplementary Fig. S3). Given that the root transcriptional response is tuned by a local nitrate signal in combination with systemic N demand (Ruffel *et al.*, 2011), we further used a split-root system, in which the roots were subjected to either homogeneous or heterogeneous nitrate supply or no N, to test whether *ZmTMM1* is under the control of local or systemic N signals. The maize seedlings subjected to the split-root culture consistently showed that *ZmTMM1* transcript levels in portions of the split roots locally fed with nitrate and the whole root system homogeneously supplied with nitrate were comparable and significantly higher than in the roots either locally or homogeneously submerged in the no N nutrient solution, suggesting that *ZmTMM1* expression is induced by local nitrate signal rather than by the systemic N status of plants (Fig. 4D). In addition, this local increase in *ZmTMM1* transcripts by nitrate mainly occurred in the LR branching zones (Fig. 4E).

### Ectopic expression of *ZmTMM1* modulates LR development in transgenic Arabidopsis

**Fig. 5.**
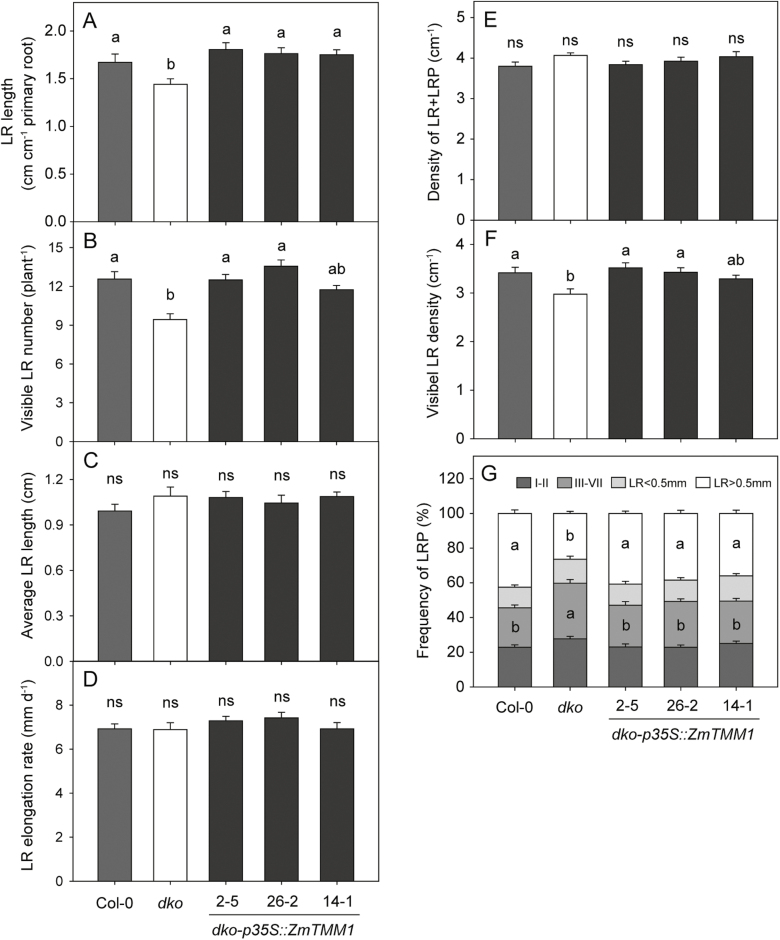
Ectopic expression of *ZmTMM1* restores LR development in the *dko* mutant. *ZmTMM1* was ectopically expressed in the Arabidopsis *dko* mutant under the control of the CaMV *35S* promoter. Arabidopsis plants were cultivated on N-free half-strength MS agar medium supplemented with 1 mM KNO_3_ as the N source, and root phenotypes were analyzed 12 d after germination. (A) LR length. (B) Visible LR number. (C) Average LR length. (D) LR elongation rate during the day 10–day 12 period presented as the mean rate of the two longest LRs. (E) Total LR density. (F) Visible LR density. (G) Relative frequency of LR primordia and emerged LRs. LR primordia and LRs at specific developmental stages were classified into four groups: stage I–II; stage III–VII; emerged LR <0.5 mm; and visible LR >0.5 mm. Bars represent means ±SE (*n*=16 replicates; each replicate represents a single seedling). Different letters represent significant differences among means at *P*<0.05; ns, not significant (Tukey’s test).

Given the overlapping gene expression patterns and redundant functions of *ANR1* and *AGL21* in LR development (Supplementary Fig. S4; Zhang and Forde, 1998; Gan *et al.*, 2012; L.H. Yu *et al.*, 2014), an Arabidopsis double mutant (*dko*) of *ANR1* and *AGL21* was generated to serve as a genetic background for functional characterization of *ZmTMM1* (Supplementary Fig. S5A). The *dko* (*anr1 agl21*) mutant showed a reduction in total LR length compared with the wild type, as explained by the decrease in the visible LR number rather than the average LR length (Supplementary Fig. S5B). Analysis of LR development by stages further indicated that a larger proportion of LR primordia were arrested in the *dko* mutant at earlier stages, leading to a relative decrease in the reduced visible LR density, while the number of LR initiation events monitored as the total density of LR and LR primordia was unaffected (Fig. 5E–G). Thus, the growth impairment of LR primordia was suggested to be the major defect in LR development identified in the *dko* mutant.

*ZmTMM1* was then ectopically overexpressed in the Arabidopsis *dko* mutant under the control of the CaMV *35S* promoter, and three independent transgenic lines (*dko-p35S::ZmTMM1*) were selected for root morphological analysis (Supplementary Fig. S6). Under a condition with uniform nitrate supply, overexpression of *ZmTMM1* recovered the LR growth of the *dko* mutant. Specifically, it restored the LR emergence in the *dko* mutant, increasing the density of visible LRs, while not altering the LR elongation rate and LR initiation (Fig. 5A–G). These results indicate that the truncated MADS-box gene *ZmTMM1* is able to complement the functions of *ANR1* and *AGL21* promoting LR development in Arabidopsis.

### Local activation of ZmTMM1 promotes LR elongation in a nitrate-dependent manner

**Fig. 6.**
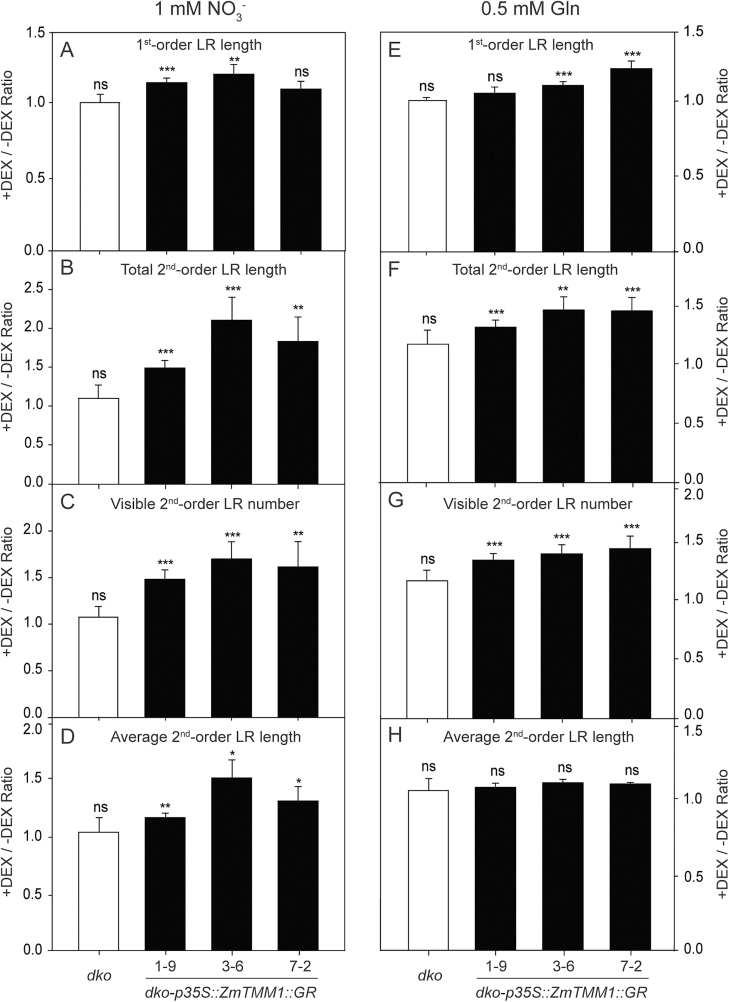
Local activation of ZmTMM1–GR fusion protein modulates LR growth. ZmTMM1–GR fusion protein expressed under the control of the CaMV *35S* promoter was locally activated by DEX supply in the split-root system. Arabidopsis seedlings harboring two LRs with similar length were transferred to vertically split agar plates containing 1 μM DEX in the +DEX side and no DEX in the –DEX side. The root phenotype was measured 8 d after transfer. (A–D) Split agar plates containing 1 mM KNO_3_ as the N source; (E–H) split agar plates containing 0.5 mM Gln as the N source. +DEX/–DEX ratio of first-order LR length (A and E); +DEX/–DEX ratio of total second-order LR length (B and F); +DEX/–DEX ratio of visible second-order LR number (C and G); +DEX/–DEX ratio of average second-order LR length (D and H). Bars represent means ±SE (*n*=7–12 replicates; each replicate represents a single seedling). Asterisks indicate significant differences between the LRs on DEX-treated (+DEX) and non-treated (–DEX) segments of agar plates at *P<0.05; **P<0.01; ***P<0.001; ns, not significant (Student’s *t*-test).

The nuclear targeting of GR fusion protein is controlled in the presence of DEX, which allows local activation of the function of transcription factor–GR fusion proteins following DEX treatment (Brockmann *et al.*, 2001; Gan *et al.*, 2012). To mimic the stimulatory effect of local nitrate supply on LR growth, a ZmTMM1–GR fusion gene construct was generated and introduced into the *dko* background (Supplementary Fig. S7B, C). In the split-root system, ZmTMM1–GR fusion protein expressed under the control of the CaMV *35S* promoter was locally activated in the DEX-containing segment (Supplementary Fig. S7D). When nitrate was supplied as the N source, the local activation of ZmTMM1–GR fusion protein promoted LR growth in the transgenic plants, increasing both the LR number and the average LR length significantly, as presented by the +DEX/–DEX ratios (Fig. 6A–D; Supplementary Fig. S7D). When Gln was supplied as an alternative N source, however, DEX-induced activation of ZmTMM1–GR fusion protein only increased the LR number but not the average LR length (Fig. 6E–H; Supplementary Fig. S7D). These results indicate that the local activation of ZmTMM1 protein in transgenic Arabidopsis promotes LR growth, stimulating LR elongation in a nitrate-dependent manner and enhancing LR branching in a nitrate-independent manner.

**Fig. 7.**
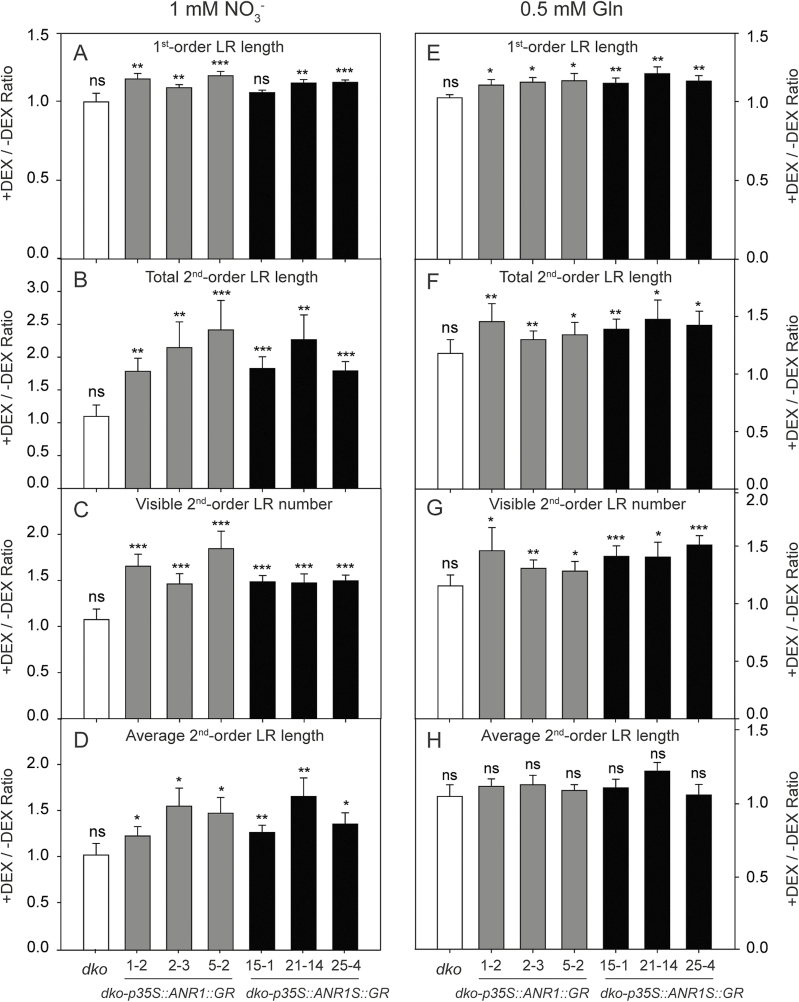
Truncated protein ANR1S functions as the intact ANR1 protein regulating LR development. ANR1–GR and ANR1S–GR fusion proteins expressed under the control of the CaMV *35S* promoter were locally activated by DEX in the split-root system. Arabidopsis seedlings harboring two LRs of similar length were transferred to vertically split agar plates containing 1 μM DEX in the +DEX side and no DEX in the –DEX side. The root phenotype was measured 8 d after transfer. (A–D) Split agar plates containing 1 mM KNO_3_ as the N source; (E–H) split agar plates containing 0.5 mM Gln as the N source. +DEX/–DEX ratio of first-order LR length (A and E); +DEX/–DEX ratio of total second-order LR length (B and F); +DEX/–DEX ratio of visible second-order LR number (C and G); +DEX/–DEX ratio of average second-order LR length (D and H). Bars represent means ±SE (*n*=7–12 replicates; each replicate represents a single seedling). Asterisks indicate significant differences between the LRs on DEX-treated (+DEX) and non-treated (–DEX) segments of agar plates at *P<0.05; **P<0.01; ***P<0.001; ns, not significant (Student’s *t*-test).

Accordingly, as ZmTMM1 is a truncated form of a MADS-box protein, the K- and C-domains were suggested to be dispensable for LR development. To verify this hypothesis, an artificially truncated form of Arabidopsis ANR1 protein (ANR1S; 93 amino acid residues), mimicking the structure of ZmTMM1, was constructed by the deletion of the K- and C-domains (Supplementary Fig. S7A, B). Then, driven by the CaMV *35S* promoter, the ANR1S–GR fusion and ANR1–GR as a positive control were expressed in the *dko* mutant (Supplementary Fig. S7B, C). It was shown that local activation of the truncated protein ANR1S and the intact protein ANR1 in the DEX-containing patches stimulated LR growth (Fig. 7). As ZmTMM1 did, both the locally activated ANR1S and ANR1 led to an increase in both the average LR length and the LR number in the presence of nitrate (Fig. 7A–D); in the absence of nitrate, however, there was only an increase in the LR number (Fig. 7E–H). These results supported that, despite the K- and C-domain truncations, ZmTMM1 can fully function as a MADS-box protein modulating LR development.

### *ZmTMM1-RNAi* transgenic maize shows no discernible phenotype compared with wild-type plants

To investigate the function of *ZmTMM1* in maize, we knocked down *ZmTMM1* transcripts by RNAi in transgenic maize. In three independent *ZmTMM1-RNAi* transgenic maize lines that we generated, the *ZmTMM1* transcript abundance decreased specifically, while the levels of its homologous gene transcripts (e.g. *ZmMADS2* and *GRMZM2G055782*) remained unchanged relative to the wild type (Supplementary Fig. S8B). In the *ZmTMM1-RNAi* lines, the induction of *ZmTMM1* transcript expression by local nitrate supply was reduced to 10–50% the levels of the wild-type plants (Supplementary Fig. S8B), but their LRs still responded to nitrate and grew to a similar extent as the wild type (Supplementary Fig. S8A, C). Consistent with these observations, the response of LR proliferation to local nitrate supply was also unaffected in the Arabidopsis *dko* mutant in the split-root system (Supplementary Fig. S5C). These results may imply the functional redundancies among the *AGL17-like* genes or other compensatory mechanisms complementing the loss of function of MADS-box proteins for root nitrate foraging.

### Induced expression by local nitrate is a common feature of *ZmTMM1-like* genes in cereals

In grass species, comparative genome analysis provides a deeper insight into the paralogous and orthologous relationships among the members of large gene families, uncovering their intraspecific and interspecific collinearity (Bolot *et al.*, 2009). In rice, among the five members of MADS-box genes in the *AGL17-like* clade, *OsMADS27* and *OsMADS61* showed an intragenomic collinearity that is caused by the intragenome duplication between chromosome 2 and chromosome 4, and *OsMADS23* revealed an intragenomic micro-collinearity with both due to a segmental duplication event. In contrast, *OsMADS25* and *OsMADS57* are likely to be the homologs that evolved independently (Fig. 2B; Supplementary Dataset S1). In addition, the interspecific collinearity of *AGL17-like* MADS-box genes was dissected in Brachypodium, sorghum, and maize by using the rice genome as a reference (Fig. 2B; Supplementary Dataset S1). Notably, *GRMZM2G344991* and *GRMZM2G052045* in maize lost the DNA-binding domain, thus they were not recognized as AGL17-like transcription factor genes. Comparative genome analysis revealed that *ZmTMM1-like* genes, comprising *ZmTMM1*, *Sb04g028960*, *BRADI3G57017*, and *OsMADS57*, independently evolved in grass species, implying that they might exhibit unique functional features.

**Fig. 8.**
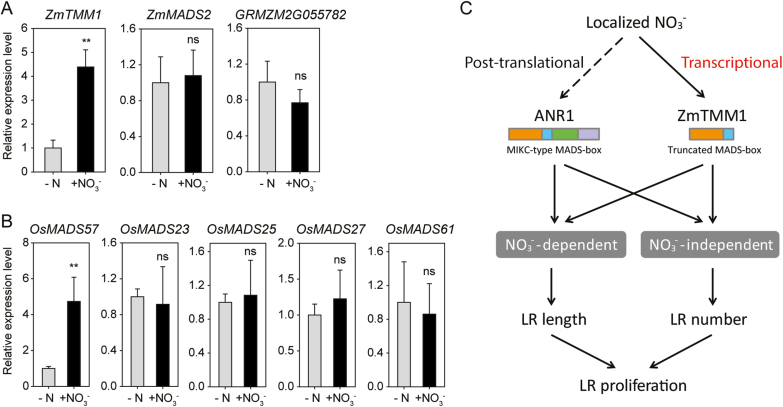
Local nitrate-inducible transcriptional regulation is a conserved feature of *ZmTMM1-like* genes. (A) Expression of maize *AGL17-like* genes in response to local nitrate supply. Maize seedlings were cultivated in a split-root system containing 1 mM KNO_3_ in the +N compartment and 0.5 mM K_2_SO_4_ in the –N compartment. After 12 h of local nitrate treatment in the split-root system, the transcript levels of target genes in roots were determined by qPCR. Values are normalized relative to maize *Tubulin 4* (*AJ420856*). (B) Expression of rice *AGL17-like* genes in response to local nitrate supply. Rice seedlings were cultivated in a split-root system as described for maize. After 12 h of local nitrate treatment, the transcript levels of target genes in roots were determined by qPCR. Values are normalized by *OsACTIN* (*LOC_Os03g50890*). Data represent means ±SD (*n*=3 replicates; each replicate represents a single seedling). Asterisks indicate significant differences between the gene expression in roots in the +N and –N compartments at: *P<0.05; **P<0.01; ns, not significant (Student’s *t*-test). (C) A model scheme describing the function and regulation of the truncated MADS-box gene *ZmTMM1*. In Arabidopsis, local nitrate activates ANR1 via a post-translational mechanism to stimulate LR proliferation; while in maize, local nitrate induces the expression of *ZmTMM1* transcripts to promote LR development.

Within the *ZmTMM1-like* clade, *BRADI3G57017* and *OsMADS57* are the complete MIKC-type MADS-box genes, while *Sb04g028960* lacks the K-domain and *ZmTMM1* is a truncated form devoid of both the K- and C-domains (Fig. 2A), suggesting that the absence of K- and C-domains is not a common feature of *ZmTMM1-like* genes in grass species. By evaluating transcript expression levels in the split-root system, *OsMADS57* and *ZmTMM1* were shown to be specifically up-regulated by local nitrate supply among all the *AGL17*-*like* genes in rice and maize (Fig. 8A, B). Thus, we propose the local nitrate-inducible expression as a conserved feature of the *ZmTMM1-like* genes in grass species. In contrast, all the *AGL17-like* genes in Arabidopsis were not regulated by local nitrate supply at the transcriptional level (Supplementary Fig. S9), suggesting that nitrate regulates *AGL17*-*like* genes via distinct mechanisms between dicots and monocots.

## Discussion

Root system architecture shows complex morphology and plasticity to adapt to nutrient heterogeneity (Giehl and von Wirén, 2014). Identification of key regulatory components modulating root morphological responses to nutrient heterogeneity is of remarkable ecological and agronomic relevance for improving nutrient use efficiency in cropping systems (Rogers and Benfey, 2015). In this study, we identified a truncated MIKC-type MADS-box transcription factor gene *ZmTMM1* involved in root nitrate-foraging responses.

### *ZmTMM1* is a unique truncated MADS-box gene modulating LR development

Genome duplications and chromosome fusions frequently occur in grass species during evolution, which reconstruct the genome and lead to functional diversification among the paralogs and orthologs (Bolot *et al.*, 2009). The *AGL17-like* gene products in dicots exhibit the complete protein structure of the classic MIKC-type MADS-box genes. In contrast, the truncated *AGL17-like* MADS-box genes are found exclusively in grass species (Fig. 2A). It is suggested that these truncated forms have evolved as a result of the insertion of an ancestral intron between the I- and K-domains (Supplementary Table S1; Supplementary Fig. S1). This intron can be removed during the mRNA splicing process, as in the case of *AGL17-like* genes in Arabidopsis and rice (Supplementary Fig. S1). However, if the size of the intron was too large for mRNA splicing, then the first half of the coding sequence only with the M- and I-domains causes a truncated MADS-box protein to be produced, although the remaining second half of the coding sequence may still exist downstream on the genome, such as those identified for *BRADI5G12440*, *Sb07g021110*, *GRMZM2G055782*, and *GRMZM2G032905* (Supplementary Table S1). In addition, not via mRNA splicing, the truncated forms can also be directly transcribed from the MADS-box genes, in which the genomic regions corresponding to the introns and the following coding sequences are completely lost presumably during the genome reconstruction, as exemplified by *ZmTMM1*, *Sb04g024010*, and *Sb06g019040* (Supplementary Table S1). It should be noted that all *AGL17-like* gene products in rice still exhibit the complete protein structure of MIKC-type MADS-box genes (Fig. 2A; Supplementary Fig. S1), indicating that the deletion of K- and C-domains might have occurred after the subdivergence of rice with other grass species.

For MIKC-type MADS-box transcription factors, the M-domain is responsible for DNA binding, the K-domain facilitates protein–protein interaction, and the C-terminal domain participates in transcriptional regulation (Kaufmann *et al.*, 2005). The C-terminal deletion of two Arabidopsis MADS-box genes, *SOC1* and *AG*, produces two truncated non-functional isoforms that act as artificial interfering peptides to inactivate the original SOC1 and AG by protein–protein interaction (Seo *et al.*, 2012). In some other cases, however, the C-terminal domain seems to be dispensable for the functions, such as rice OsMADS57 in control of tillering (Guo *et al.*, 2013) and Arabidopsis AP3 and PI in flowering regulation (Piwarzyk *et al.*, 2007; Su *et al.*, 2008). Thus, the MADS-box domain and I-domain appear to form the minimum unit responsible for DNA binding, which is essential for the specificity of MICK-type MADS-box transcription factors (Kaufmann *et al.*, 2005). In this study, ZmTMM1 containing only the M- and I-domains has been shown to be a functional MADS-box transcription factor (Fig. 2A). ZmTMM1–GFP fusion protein is located in the nucleus, and both the overexpression of ZmTMM1 and the DEX-mediated local activation of ZmTMM1–GR fusion protein recovered LR development of the Arabidopsis *dko* mutant (Figs 5, 6). The GR fusion of an artificially truncated ANR1S protein, mimicking the protein structure of ZmTMM1, also promotes the LR growth similar to the way in which the intact ANR1 protein does in the *dko* background (Fig. 7), implicating that K- and C-domains are not essential for the function of *AGL17-like* genes to regulate LR development.

*AGL17-like* MADS-box genes confer a conserved function in LR development, as indicated by roles of *ANR1* and *AGL21* in Arabidopsis and *OsMADS25* and *OsMADS57* in rice (Zhang and Forde, 1998; Gan *et al.*, 2012; L.H. Yu *et al.*, 2014; C. Yu *et al.*, 2015; Zhang *et al.*, 2018; Huang *et al.*, 2019). Among them, *ANR1*, *OsMADS25*, and *OsMADS57* are involved in root nitrate foraging. Ectopic overexpression of *ZmTMM1* in the Arabidopsis *dko* mutant revealed that *ZmTMM1* stimulates LR growth, as is reflected, respectively, by the nitrate-dependent and -independent increase in the LR length and the LR number (Figs 6, 7; Gan *et al.*, 2012). A monocot-exclusive miRNA, miR444, additionally regulates *AGL17-like* genes (*OsMADS23*, *OsMADS27*, and *OsMADS57*) in rice by targeting the coding sequence in the K-domain, which leads to post-transcriptional degradation of the coding mRNA (Guo *et al.*, 2013; Yan *et al.*, 2014). The lack of a K- and C-domain in *ZmTMM1* and other *ZmTMM1-like* truncated MADS-box genes in grass species thus may imply a strategy evolved to escape from the negative control mediated by miR444. The functional conservation of *AGL17-like* genes in LR development also implies the existence of genetic redundancy among the homologs. Indeed, the Arabidopsis *anr1* single mutant and the *anr1 agl21* double mutant showed local nitrate-induced LR proliferation to a similar extent as the wild type (Gan *et al.*, 2005; Supplementary Fig. S5C). Similarly, in maize, the response of LR growth to local nitrate was not affected in the *ZmTMM1-RNAi* lines (Supplementary Fig. S8), suggesting a possible functional compensation because of the presence of the remaining *AGL17-like* genes such as *ZmMADS2* and *GRMZM2G055782*. Since these two genes did not respond to local nitrate supply at the transcript expression levels (Fig. 8A), their post-translational regulation can be postulated as a mechanism that could have compensatorily stimulated LR proliferation in nitrate-fed root segments in the maize *ZmTMM1-RNAi* lines (Supplementary Fig. S8) as ANR1 does in Arabidopsis (Fig. 8C). Further studies on multiple knockout or overexpression lines may be necessary to fully understand the function of *AGL17-like* genes in maize.

### Local nitrate induction is a conserved feature of *ZmTMM1-like* genes in cereals

Root-preferential expression is an ancestral feature of the *AGL17-like* genes (Becker and Theissen, 2003). In Arabidopsis, three of the *AGL17*-like members (*ANR1*, *AGL21*, and *AGL17)* are mainly expressed in root tips despite their differential cell type specificity of gene expression. For example, *ANR1* and *AGL21* are preferentially expressed in the LR primordia and central cylinder of mature roots, while *AGL17* is specifically detected in the LR cap and the epidermis (Supplementary Fig. S4; Burgeff *et al.*, 2002; Remans *et al.*, 2006; L.H. Yu *et al.*, 2014). In rice, the expression of *OsMADS23*, *OsMADS25*, *OsMADS27*, and *OsMADS57* is detected in the root central cylinder (Puig *et al*, 2013); in particular, *OsMADS57* expression is shown in the xylem parenchyma cells (Huang *et al*, 2019). *ZmTMM1* transcripts are also found in the xylem parenchyma cells of mature roots and the cortical cells surrounding the LR base (Fig. 3). Thus, during evolution, both the intact and truncated forms of *AGL17-like* genes, either in dicots or in monocots, may have retained the root-preferential expression pattern from their ancestor, supporting their conserved roles in root nutrient foraging.

However, *AGL17-like* genes in dicot and monocot species show distinct responses to N availability. In Arabidopsis, *ANR1*, *AGL21,* and *AGL16* are up-regulated by N deprivation and repressed by N resupply, while *AGL17* is unaffected by the changes in N availability (Gan *et al.*, 2005). In the split-root system, the expression of all these Arabidopsis *AGL17-like* genes is not transcriptionally regulated by local supply of nitrate (Supplementary Fig. S9). To stimulate LR proliferation in a nitrate-rich zone, ANR1 is proposed to be subject to a post-translational regulation in the presence of nitrate (Gan *et al.*, 2012). In contrast, in rice roots, *OsMADS25*, *OsMADS27*, and *OsMADS57* are transcriptionally induced in response to nitrate supply (Puig *et al.* 2013; C. Yu *et al.*, 2014; Huang *et al.*, 2019). It should be noted that previous studies reporting the expression patterns of *AGL17-like* genes in Arabidopsis or rice were conducted under conditions of homogenous nitrate supply, not allowing differentiation of their regulation via local or systemic nitrate signaling pathways (Gan *et al.*, 2005; Puig *et al.* 2013; Li *et al.*, 2014; C. Yu *et al.*, 2014; Huang *et al.*, 2019). Therefore, in the present study, we performed split-root experiments to investigate the response of *AGL17-like* genes to local N signaling specifically (Figs 4C, D, 8A, B; Supplementary Fig. S9). Unlike other *AGL17-like* genes, *ZmTMM1-like* genes in the monocot-specific subclade are transcriptionally regulated by local nitrate rather than a systemic N signal. This expression pattern suggests that *ZmTMM1-like* genes are the homologs that could have retained the transcriptional control mechanisms relevant to responses to local nitrate supply even after the divergence of dicot and monocot species. It may be speculated that the two key features of *ZmTMM1*, truncated protein and transcriptional regulation by local nitrate, are likely to have evolved independently.

Taken together, we propose a model whereby in the dicot plant Arabidopsis, local supply of nitrate activates ANR1 at the post-translational level to stimulate LR proliferation; however, in grass species such as maize, local supply of nitrate directly induces the expression of *ZmTMM1* at the transcriptional level to promote LR development in nitrate-fed root segments (Fig. 8C). The post-translational control may also exist in ZmTMM1, although it is a truncated MADS-box gene. This study provides new insights into diverse functions and regulations of MICK-type MADS-box genes across different plant species, and opens up an avenue toward understanding of developmental control mechanisms involved in root nitrate foraging.

## Supplementary data

Supplementary data are available at *JXB* online.

Fig. S1. The exon–intron structure of *AGL17-like* genes in Arabidopsis and rice.

Fig. S2. Root-preferential expression of *ZmTMM1* in maize.

Fig. S3. Expression of an N-responsive marker gene *ZmGS1.1* and total N concentration in maize roots in response to local N supply in a split-root system.

Fig. S4. Localization of *AGL21* and *ANR1* promoter activities in Arabidopsis roots.

Fig. S5. LR growth phenotype of the Arabidopsis *dko* mutant.

Fig. S6. Ectopic expression of *ZmTMM1* in the *dko* mutant.

Fig. S7. Construction and analysis of ZmTMM1–, ANR1S–, and ANR1–GR fusion transgenic lines.

Fig. S8. Phenotypic analysis of *ZmTMM1-RNAi* transgenic maize under local nitrate supply.

Fig. S9. Expression of Arabidopsis *AGL17-like* genes in response to local nitrate supply.

Table S1. Gene structure of truncated *AGL17-like* genes and orthologs in monocots identified from comparative genome analysis.

Table S2. Primers used in this study.

Dataset S1. Comparative genome analysis of *AGL17-like* genes in monocots.

eraa116_suppl_Supplementary_Material

eraa116_suppl_Supplementary_Dataset
