# Supplementary material for: Involvement of a truncated MADS-box transcription factor ZmTMM1 in root nitrate foraging
Source: J Exp Bot. 2020 Mar 5;71(15):4547–61. doi: 10.1093/jxb/eraa116 (PMC7382388; doi:10.1093/jxb/eraa116)
Supplement: eraa116_suppl_Supplementary_Material [file eraa116_suppl_supplementary_material.pdf]

Supplementary Data

Involvement of a truncated MADS-box transcription factor *ZmTMM1* in root nitrate foraging

Ying Liu[1], Zhongtao Jia[1], Xuelian Li[1], Zhangkui Wang[1], Fanjun Chen[1], Guohua Mi[1], Brian Forde[2], Hideki Takahashi[3], Lixing Yuan[1,4*]

[1] Key Lab of Plant-Soil Interaction, MOE, College of Resources and Environmental Sciences, China Agricultural University, 100193 Beijing, China

[2] Lancaster Environment Centre, Lancaster University, LA1 4YQ Lancaster, UK

[3] Department of Biochemistry and Molecular Biology, Michigan State University, East Lansing, MI 48824, USA

[4] Center for Crop Functional Genomics and Molecular Breeding, China Agricultural University, 100193 Beijing, China

[*] Corresponding author: Lixing Yuan (yuanlixing@cau.edu.cn)

Supplementary Data

Fig. S1: The exon-intron structure of *AGL17-like* genes in Arabidopsis and rice.

Fig. S2: Root preferential expression of *ZmTMM1* in maize.

Fig. S3: Expression of a N-responsive marker gene *ZmGS1.1* and total N concentration in maize roots in responses to local N supply in split-root system.

Fig. S4: Localization of *AGL21* and *ANR1* promoter activities in Arabidopsis roots.

Fig. S5: LR growth phenotype of Arabidopsis *dko* mutant.

Fig. S6: Ectopic expression of *ZmTMM1* in the *dko* mutant.

Fig. S7: Construction and analysis of ZmTMM1-, ANR1S- and ANR1-GR fusion transgenic lines.

Fig. S8: Phenotypic analysis of *ZmTMM1-RNAi* transgenic maize under local nitrate supply.

Fig. S9: Expression of Arabidopsis *AGL17*-like genes in response to local nitrate supply.

Table S1: Gene structure of truncated *AGL17-like* genes and orthologs in monocots identified from comparative genome analysis.

Table S2: Primers used in this study.

Dataset S1: Comparative genome analysis of *AGL17-like* genes in monocots.

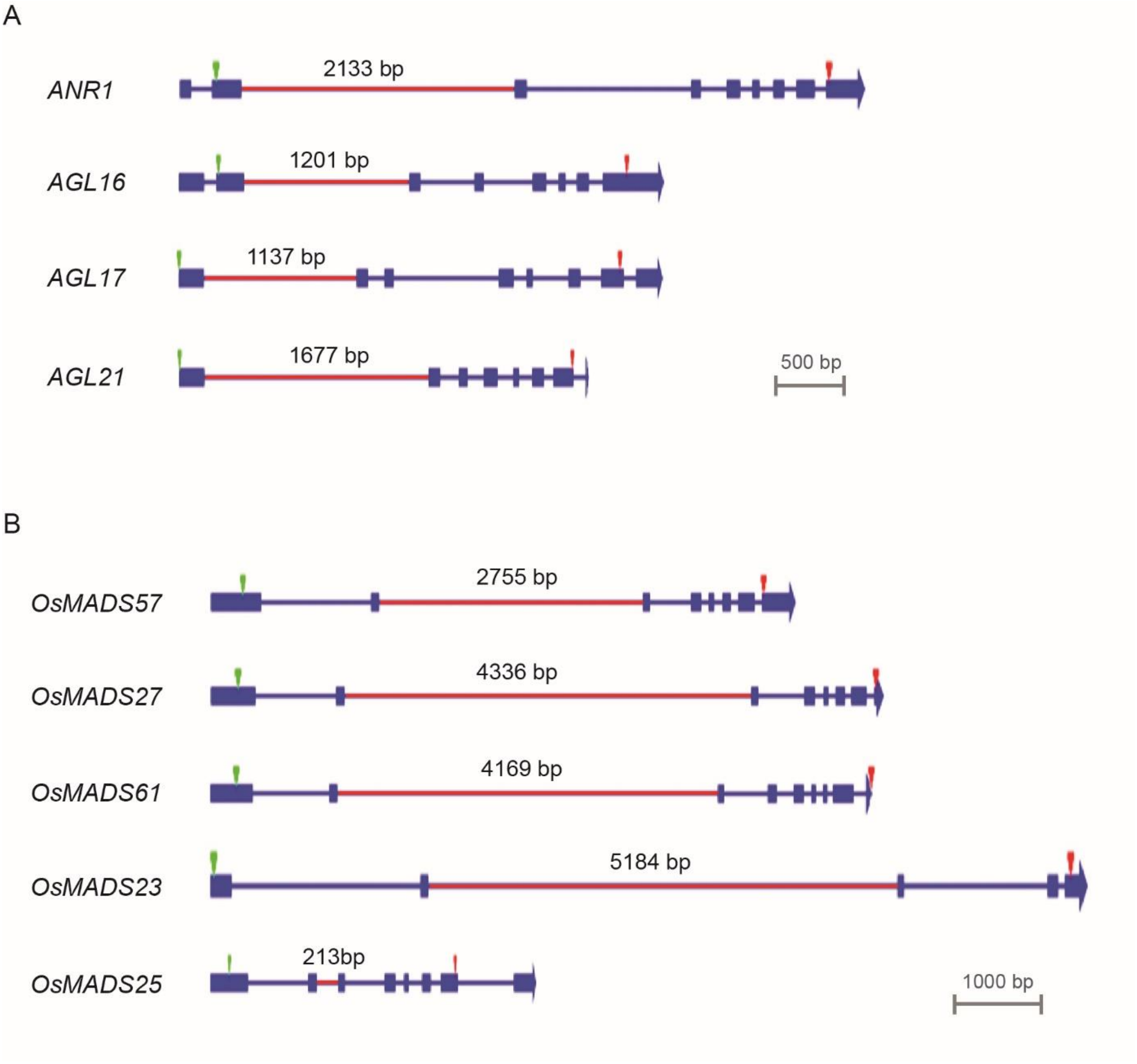

Supplementary Fig. S1. The exon-intron structure of *AGL17-like* genes in Arabidopsis and rice

Gene structure of *AGL17-like* genes in Arabidopsis (A) and rice (B). Gene structure annotation was conducted by yrGATE algorithm. Blue blocks stand for exons and blue lines for introns. Magenta-colored lines highlight the intron inserted into the gap between I- and K- domain of MIKC-type MADS-box genes. Green and red arrow heads indicate the site of start and stop codons, respectively.

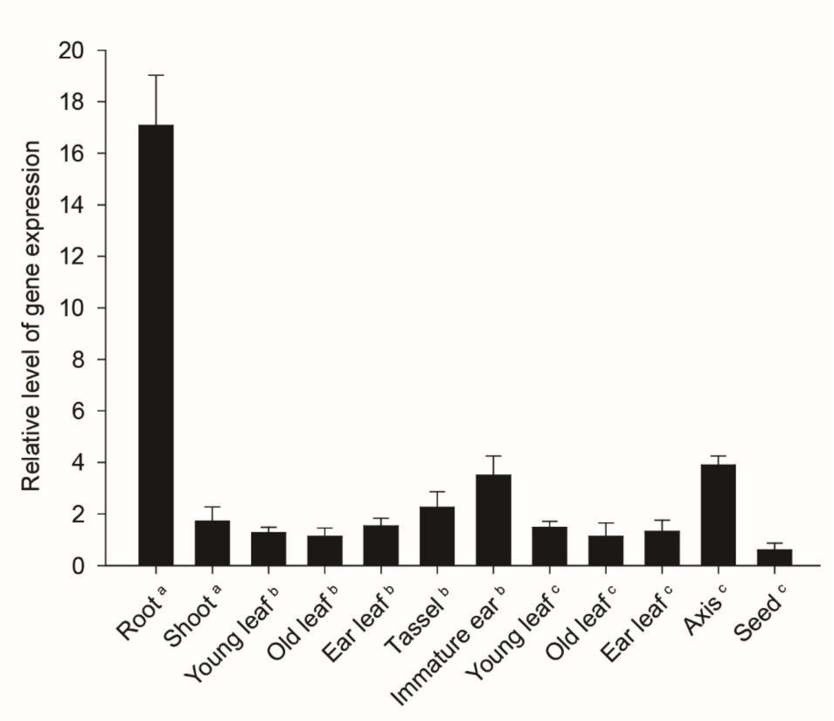

Supplementary Fig. S2. Root preferential expression of *ZmTMM1* in maize

Expression level of *ZmTMM1* transcript was analyzed by quantitative real-time PCR and normalized by maize *GAPDH (gi22302)*. [a] Samples were collected from field-grown maize plants at seedling stage (28 days after germination), [b] at silking stage, and [c] at stage of 15 days after pollination. Data represent means ± SD (n = 3 replicates; each replicate represents a single seedling).

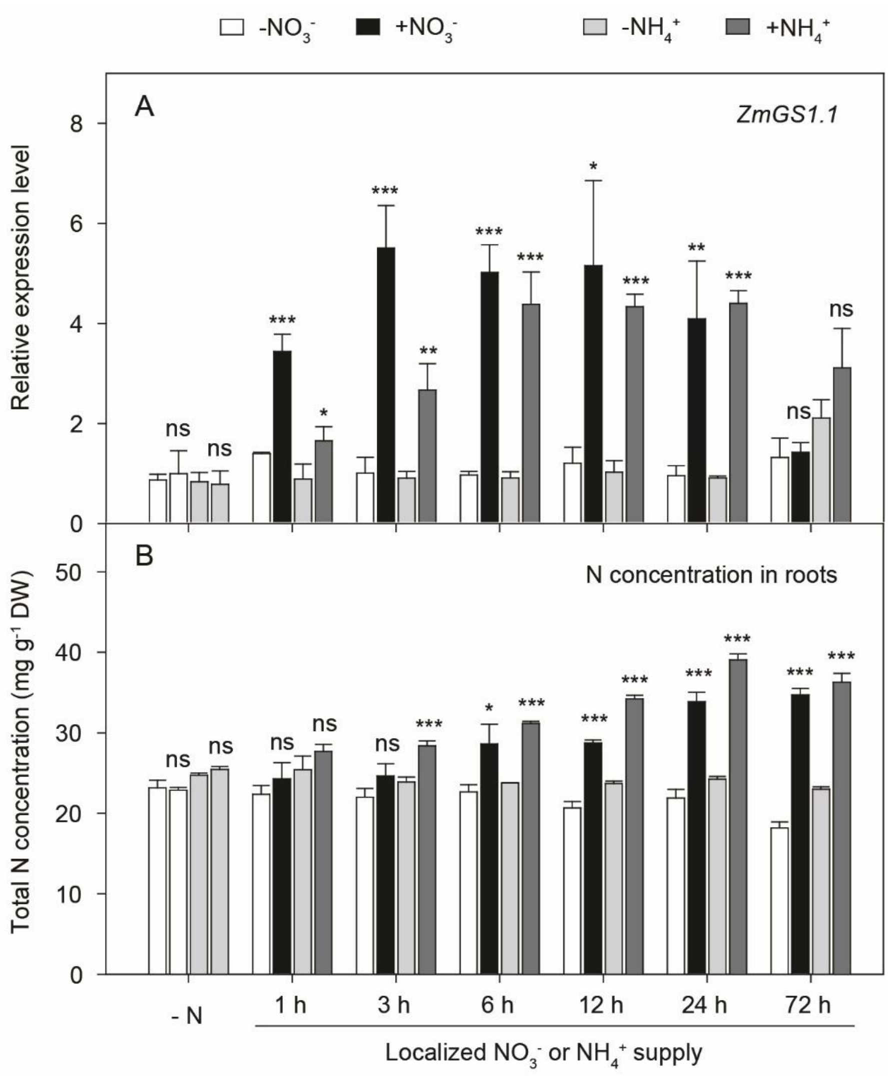

Supplementary Fig. S3. Expression of a N-responsive marker gene *ZmGS1.1* and total N concentration in maize roots in responses to local N supply in split-root system

After a N starvation for 3 days, maize seedlings with four crown roots were transferred to a split-root system containing either 1 mM KNO_3 or 0.5 mM $(NH_4)_2SO_4$ in the +N compartment, and 0.5 mM K_2SO_4 in the -N compartment, respectively. Roots were sampled for gene expression and total nitrogen concentration analysis. (A) Relative expression level of *ZmGS1.1* determined by qPCR and normalized by maize *Tubulin 4 (AJ420856)*. (B) Total N concentration in the roots. Data represent means ± SD (n = 3 replicates; each replicate represents a single seedling). Asterisks indicate significant differences between the values detected in root samples from the +N and -N compartments at *, $p < 0.05$; **, $p < 0.01$; ***, $p < 0.001$; ns, not significant (Student's *t*-test).

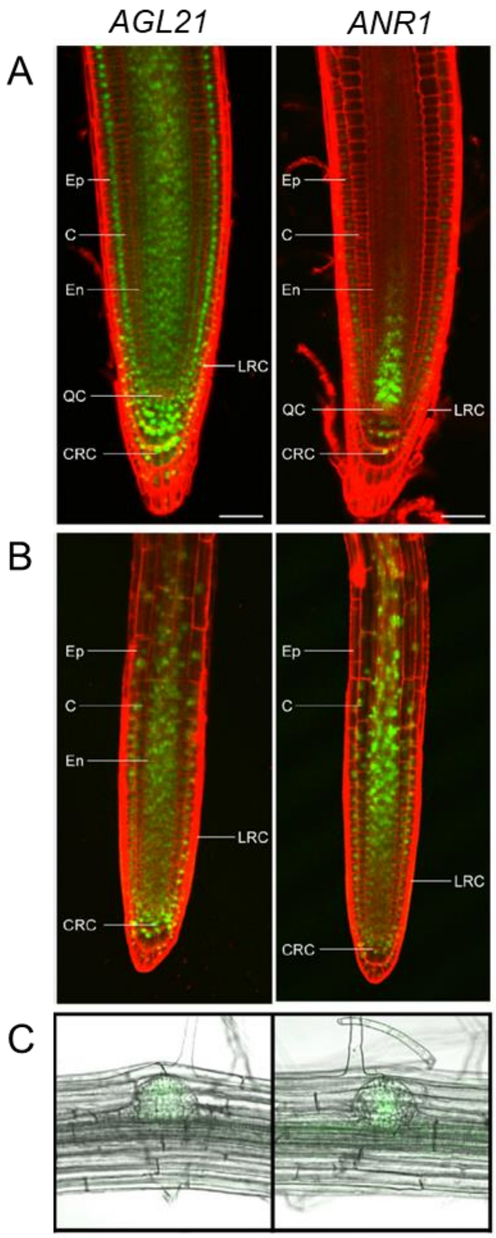

Supplementary Fig. S4. Localization of *AGL21* and *ANR1* promoter activities in Arabidopsis roots

The transgenic Arabidopsis plants expressing *AGL21 promoter-GFP-NLS* and *ANR1 promoter-GFP-NLS* were grown on agar plates supplied with nitrate for observation of GFP (green) using a confocal microscope. (A) PR tips; (B) LR tips; (C) LR primordia. Roots were counter-stained by propidium iodide (red in A and B). Ep: epidermis; C: cortex; En: endodermis; LRC: lateral roots cap; QC: quiescent center; CRC: columella cell of root caps.

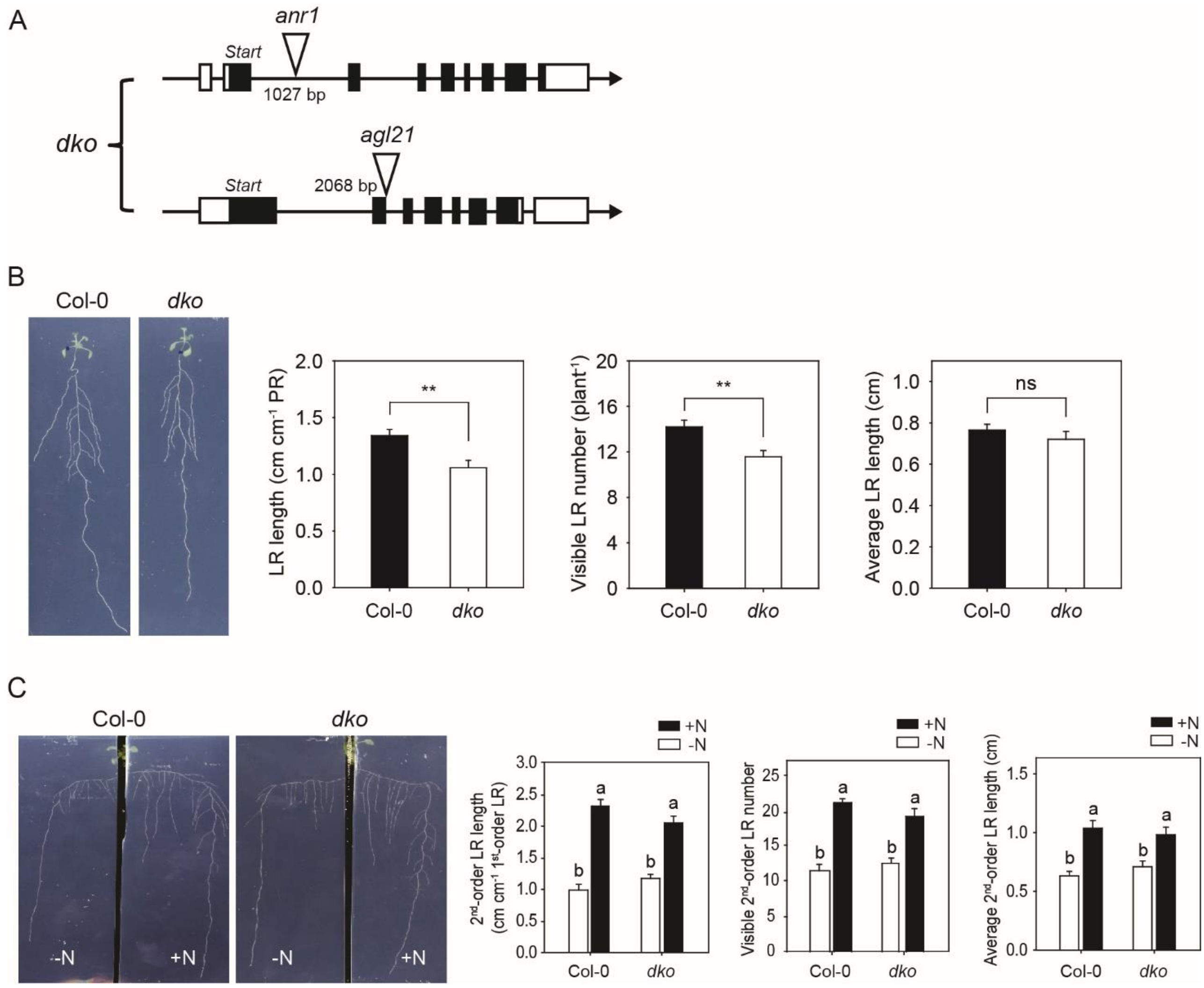

Supplementary Fig. S5. LR growth phenotype of Arabidopsis *dko* mutant.

(A) Schematic diagram of *dSpm* transposon insertions in *anr1* and *agl21* single mutants used for generating *anr1 agl21* (*dko*) mutant.

(B) Root growth of Col-0 and *dko* plants under homogenous nitrate supply. Plants were grown on N-free half-strength MS agar plates supplemented with 1 mM KNO_3 for 12 days. Bars represent means ± SE (n = 16 replicates; each replicate represents a single seedling). Asterisk indicates significant differences between *dko* and Col-0 at *, $p < 0.05$; **, $p < 0.01$; ***, $p < 0.001$; ns, not significant (Student's *t*-test).

(C) Root response of Col-0 and *dko* mutant to localized nitrate supply in vertically split segmented agar plates. Arabidopsis seedlings pruned to have only two first-order LRs were transferred to split agar plates containing 1 mM KNO_3 in the +N patch and 0.5 mM K_2SO_4 in the -N patch, respectively. Root phenotypes were measured 6 days after transfer. Bars represent means ± SE (n = 12 replicates;

each replicate represents a single seedling). Different letters represent significant differences among means at $P < 0.05$ (Tukey's test).

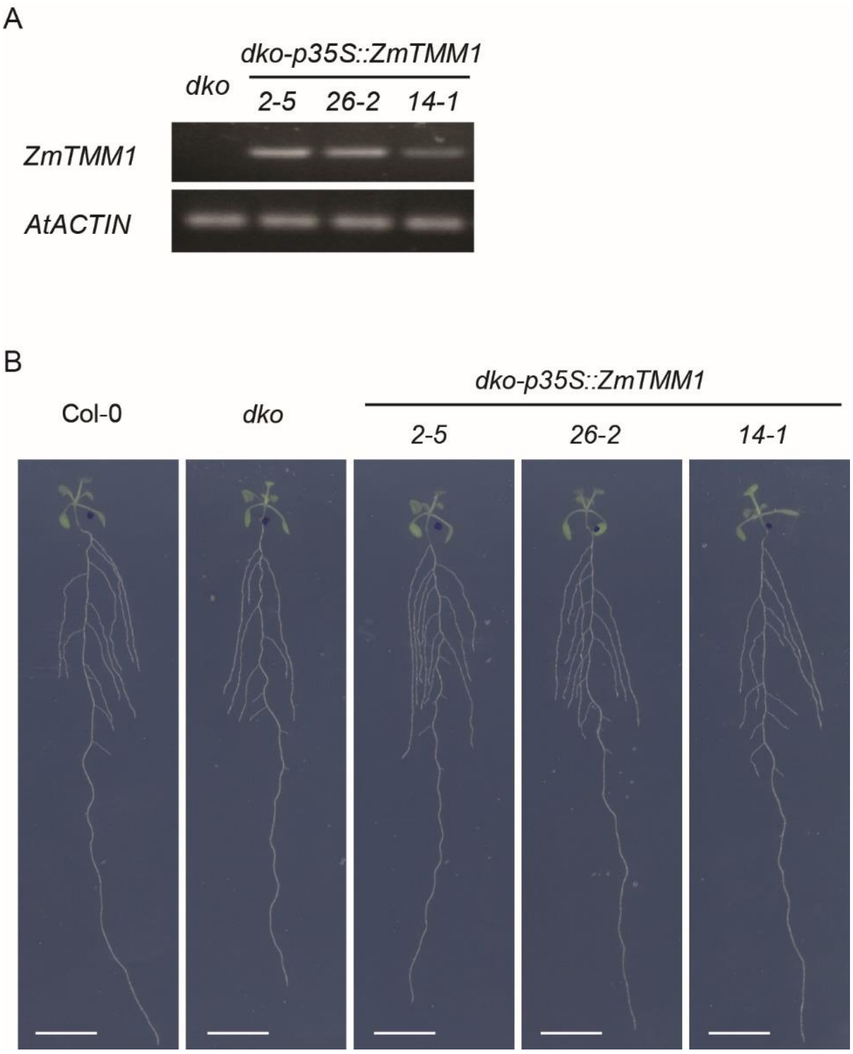

Supplementary Fig. S6. Ectopic expression of *ZmTMM1* in *dko* mutant

(A) Ectopic expression of *ZmTMM1* in *dko* mutant. Driven by *CaMV 35S* promoter, *ZmTMM1* was overexpressed in Arabidopsis *dko* mutant, and three transgenic lines were selected, named 2-5; 26-2; 14-1, respectively. Transcripts of *ZmTMM1* in transgenic lines were detected by RT-PCR. *ACTIN (AT3G18780)* was used as the internal control.

(B) Root growth of Col-0, *dko and ZmTMM1*-overexpressing lines under homogenous nitrate supply. Plants were grown on N-free half-strength MS agar plate supplemented with 1 mM KNO_3 for 12 days. Bars = 1 cm.

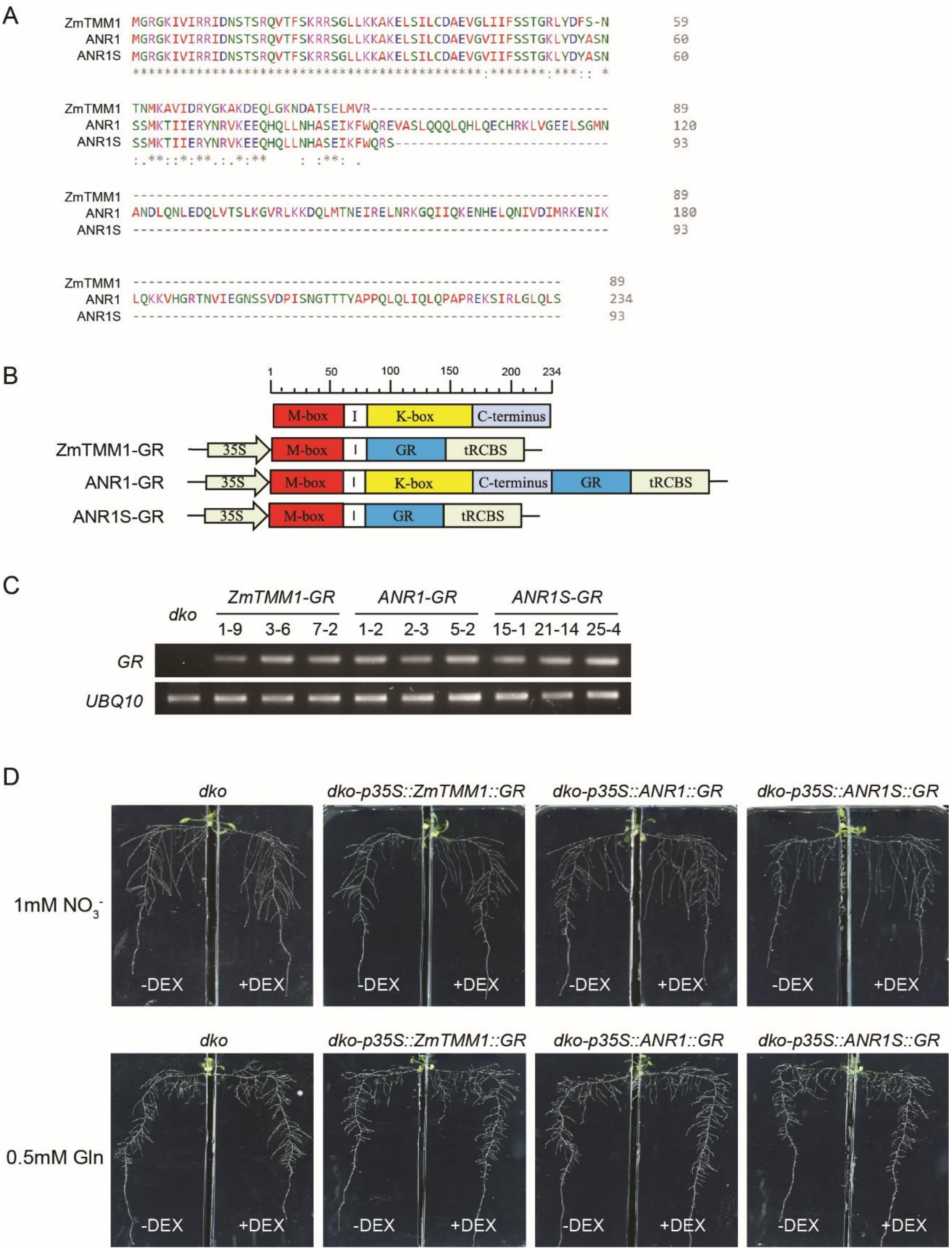

Supplementary Fig. S7. Construction and analysis of ZmTMM1-, ANR1S- and ANR1-GR fusion transgenic lines

(A) Sequence aligment of ZmTMM1, intact ANR1 and truncated ANR1S proteins.

(B) Organization of ZmTMM1-, ANR1S- and ANR1-GR fusion gene constructs.

(C) Expression of *ZmTMM1-GR, ANR1-GR*, *ANR1S-GR* transcripts in transgenic Arabidopsis plants

determined by RT-PCR.

(D) ZmTMM1-GR, ANR1-GR and ANR1s-GR fusion lines grown on local DEX supplied plates. Vertically splitted agar plates contained N-free half-strength MS medium supplemented with 1 mM nitrate or 0.5 mM Gln as N the sources. Arabidopsis seedlings harboring two 1st-order LRs were transferred to split root plates containing 1 µM DEX in the +DEX side and no DEX in the -DEX side. Root images were taken 8 days after transfer.

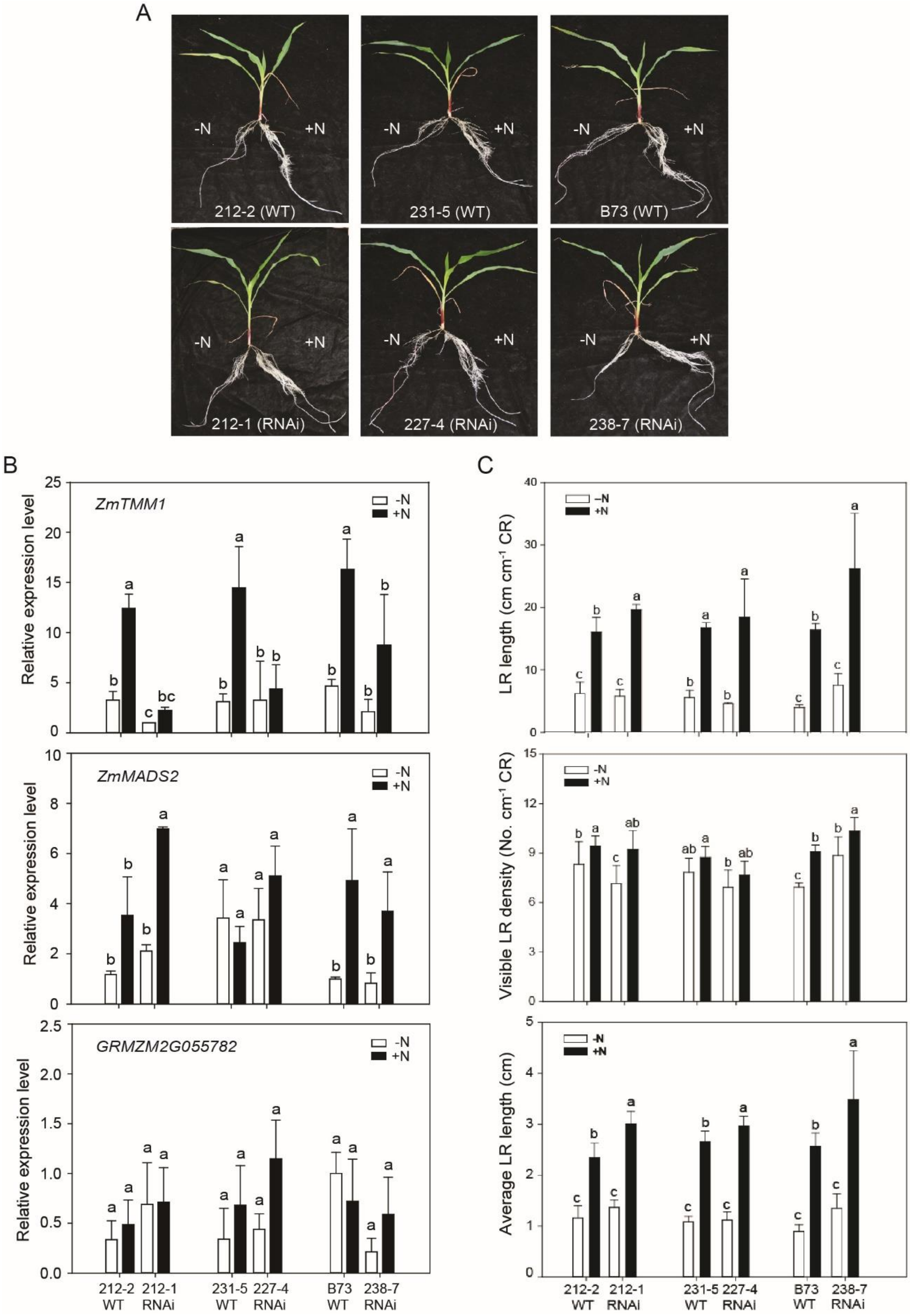

Supplementary Fig. S8. Phenotypic analysis of *ZmTMM1-RNAi* transgenic maize under local nitrate supply

Three independent *ZmTMM1-RNAi* transgenic maize lines and the corresponding wild-types (WT)

with four crown roots were cultivated in a split-root system containing 1 mM KNO_3 in the +N compartment and 0.5 mM K_2SO_4 in the -N compartment, respectively.

(A) Phenotypes of *ZmTMM1-RNAi* transgenic lines under local nitrate supply in split-root systems. Photographs were taken at 5 days after transfer. (B) *ZmTMM1*, *ZmMADS2* and *GRMZM2G055782* transcript levels in response to local nitrate supply in WT and *ZmTMM1-RNAi* lines. Gene expression was detected 12h after the transfer of seedlings to local nitrate treatment using split-root systems. Relative transcript levels of indicated genes were determined by qPCR and normalized by maize *Tubulin 4 (AJ420856)*. Data represent means ± SD (n = 4 replicates; each replicate represents a single seedling). Different letters indicate significant differences between the four bars in each group at $P < 0.05$ (Tukey's test). (C) LR development of WT and *ZmTMM1-RNAi* lines in response to local nitrate supply. Root growth were measured at 5 days after the transfer to the split-root system. Data represent means ± SD (n = 4 replicates; each replicate represents two seedlings). Different letters indicate significant differences between the four bars in each group at $P < 0.05$ (Tukey's test).

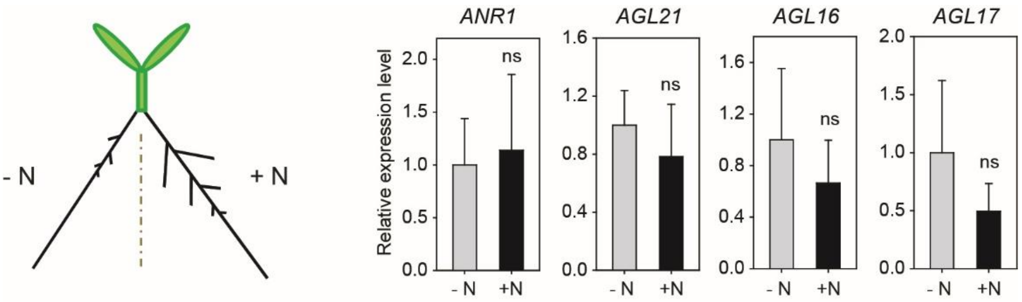

Supplementary Fig. S9. Expression of Arabidopsis *AGL17-like* genes in response to local nitrate supply.

Arabidopsis seedlings were cultivated in a split-root system which contained 1 mM KNO_3 in the +N patch and 0.5 mM K_2SO_4 in the -N patch. After 12 h of local nitrate treatment in the split-root system, transcipt levels of *AGL16*, *AGL17*, *AGL21* and *ANR1* in roots were determined by qPCR and normalized by *AtUBQ10.* Data represent means ± SD (n = 3 replicates; each replicate represents a single seedling). Asterisks indicate significant differences between the gene expression in roots on +N and -N patches at: *, $p < 0.05$; **, $p < 0.01$; ns, not significant (Student's *t*-test).

Supplementary Table S1. Gene structure of truncated *AGL17-like* genes and orthologs in monocots identified from comparative genome analysis

Species	Gene name	Protein structure	Protein length	First half on genome	Second half on genome	Length of the intron or gap
Zea mays	*ZmTMM1*	Truncated protein	89 aa	GRMZM2G044408, Chr 5	-	-
Zea mays	*ZmMADS2*	Complete protein	240 aa	GRMZM2G316366, Chr 5	GRMZM2G492156, Chr 5	5477 bp
Zea mays	*GRMZM2G055782*	Truncated protein	91 aa	GRMZM2G055782, Chr 4	GRMZM2G133568, Chr 4	21253 bp
Zea mays	*GRMZM2G032905*	Truncated protein	89 aa	GRMZM2G032905, Chr 2	GRMZM2G033093, Chr 2	20902 bp
Oryza sativa	*OsMADS61*	Complete protein	246 aa	LOC_Os04g38770, Chr 4	LOC_Os04g38780, Chr 4	4169 bp
Brachypodium distachyon	*BRADI5G12440*	Truncated protein	91 aa	BRADI5G12440, Chr 5	BRADI5G12450, Chr 5	14428 bp
Sorghum bicolor	*Sb04g024010*	Truncated protein	91 aa	Sb04g024010, Chr 4	-	-
Sorghum bicolor	*Sb06g019040*	Truncated protein	95 aa	Sb06g019040, Chr 6	-	-
Sorghum bicolor	*Sb07g021110*	Truncated protein	73 aa	Sb07g021110, Chr 7	Sb07g021100, Chr 7	9402 bp

Data source: Sequence information of *AGL17-like* genes in *Oryza sativa*, *Brachypodium distachyon*, *Sorghum bicolor*, and *Zea mays* were obtained from PlantGDB.

Supplementary Table S2. Primers used in this study

Primer name	Primer sequence	Purpose
qZmTMM1-F	5'-GGCAAGAACGATGCAACTTCAGA-3'	qRT-PCR
qZmTMM1-R	5'-CCGACCAACATACATCATATGGCA-3'	qRT-PCR
qZmMADS2-F	5'-GCAACTTGAACTCAGCACACTGC-3'	qRT-PCR
qZmMADS2-R	5'-CCTTCAGCATCATAAGACGGCAG-3'	qRT-PCR
qGRMZM2G055782-F	5'-GCTCTAGATAAATAAATCCCTCTGT-3'	qRT-PCR
qGRMZM2G055782-R	5'-CAAGATGAATGCAAAAACATATAGC-3'	qRT-PCR
qZmGS1.1-F	5'-GGTTTTGAAATTTAGATTCCGTTGTCC-3'	qRT-PCR
qZmGS1.1-R	5'-GCCAAACGTACATCTATGACGCAAG-3'	qRT-PCR
qZmGAPDH-F	5'-CTGGTTTCTACCGACTTCCTTG-3'	qRT-PCR
qZmGAPDH-R	5'-CGGCATACACAAGCAGCAAC-3'	qRT-PCR
qZmTUB4-F	5'-GCTATCCTGTGATCTGCCCTGA-3'	qRT-PCR
qZmTUB4-R	5'-CGCCAAACTTAATAACCCAGTA-3'	qRT-PCR
qOsMADS57-F	5'-CAGGCCTGTGAACAGAGAGG-3'	qRT-PCR
qOsMADS57-R	5'-CTGGAGCAGCTGTTTTGCTG-3'	qRT-PCR
qOsMADS23-F	5'-AAGCAGGAAGGTTGTTACCACT-3'	qRT-PCR
qOsMADS23-R	5'-TCCTGATGTATGAGGCTTCCCT-3'	qRT-PCR
qOsMADS27-F	5'-GACCAAGTGAAGTCAATCGGGA-3'	qRT-PCR
qOsMADS27-R	5'-AACCCTAGCTTAGGAGCAGTTG-3'	qRT-PCR
qOsMADS61-F	5'-GATTGATCGATATGGGCGAGCT-3'	qRT-PCR
qOsMADS61-R	5'-ATGAAGACGCTACAAAACTCCTTC-3'	qRT-PCR
qOsMADS25-F	5'-AACATTGCTCATCAACGCAACA-3'	qRT-PCR
qOsMADS25-R	5'-AATCACGGGGTGTACTCGATTC-3'	qRT-PCR
qOsACTIN-F	5'-TTATGGTTGGGATGGGACA-3'	qRT-PCR
qOsACTIN-R	5'-AGCACGGCTTGAATAGCG-3'	qRT-PCR
qANR1-F	5'-TTGGCAAAGAGAGGTTGCAAG-3'	qRT-PCR
qANR1-R	5'-TCTCCCACTAGTTTCCTGTGGC-3'	qRT-PCR
qAGL16-F	5'-CCACGAAGGCTCAATTCAAGCTGA-3'	qRT-PCR
qAGL16-R	5'-AAACCACCGGCAAATGATGGGG-3'	qRT-PCR
qAGL17-F	5'-TGCCAGCTCCAGTGTGAAATC-3'	qRT-PCR
qAGL17-R	5'-TTGCTCCTCCATCTTAGCCGT-3'	qRT-PCR
qAGL21-F	5'-CTTCATGCTGGAGCTTGCAAAGTC-3'	qRT-PCR
qAGL21-R	5'-AGCTATTCTCTGTGATGCCGAGGT-3'	qRT-PCR
qAtUBQ10-F	5'-CTTCGTCAAGACTTTGACCG-3'	qRT-PCR
qAtUBQ10-R	5'-CTTCTTAAGCATAACAGAGACGAG-3'	qRT-PCR
qAtACTIN2-F	5'-GACCAGCTCTTCCATCGAGAA-3'	qRT-PCR
qAtACTIN2-R	5'-CAAACGAGGGCTGGAACAAG-3'	qRT-PCR
ZmTMM1-in situ-antisense	5'-TAATACGACTCACTATAGGAGGC AAGAACGATGCAACTTCAGA-3'	In situ hybridization
ZmTMM1-in situ-sense	5'-TAATACGACTCACTATAGGACCGA CCAACATACATCATATGGCA-3'	In situ hybridization

ZmTMM1-OX-F	5'-GGAGCCATGGGGAGGGGGAAGATAGT-3'	p35S::ZmTMM1 construct
ZmTMM1-OX-R	5'-ACACACGTGTCACCGTACCATGAGTTCT-3'	p35S::ZmTMM1 construct
ZmTMM1-GFP-F	5'-AGAGACTAGTATGGGGAGGGGGAAGATAGT-3'	p35S::ZmTMM1-GFP construct
ZmTMM1-GFP-R	5'-TTGTGGTACCCGTACCATGAGTTCTGAAGT-3'	p35S::ZmTMM1-GFP construct
ZmTMM1-RNAi-F1	5'-CGCGGATCCTTTCTCCAACACCAACATG-3'	pUBQ::ZmTMM1-RNAi construct
ZmTMM1-RNAi-R1	5'-CGGGGTACCCACGTGTGTACTACGACCA-3'	pUBQ::ZmTMM1-RNAi construct
ZmTMM1-RNAi-F2	5'-CGCGAGCTCTTTCTCCAACACCAACATG-3'	pUBQ::ZmTMM1-RNAi construct
ZmTMM1-RNAi-R2	5'-TGGACTAGTCACGTGTGTACTACGACCA-3'	pUBQ::ZmTMM1-RNAi construct
ZmTMM1-GR-F	5'-AGGGCCCATGGGGAGGGGGAAGATAGT-3'	p35S::ZmTMM1-GR construct
ZmTMM1-GR-R	5'-TCGTACGGTACCATGAGTTCTGAAGTTGCA-3'	p35S::ZmTMM1-GR construct
ANR1-GR-F	5'-AGGGCCCATGGGGAGAGGGAAGATAGTTA-3’	p35S::ANR1-GR construct
ANR1-GR-R	5'-TCGTACGAAAGTTGTAGCCCTAGTCTGAT-3'	p35S::ANR1-GR construct
ANR1S-GR-F	5'-AGGGCCCATGGGGAGAGGGAAGATAGTTA-3’	p35S::ANR1S-GR construct
ANR1S-GR-R	5'-ACGTACGATCTTTGCCAAAACTTTATCTCTG-3'	p35S::ANR1S-GR construct
GR-F	5'-CAGACGTACGATCCAATTCAGCAAGCC-3'	GR amplification
GR-R	5'-CTGGGTCGACTTCTTAGTAAGGCAGTC-3'	GR amplification
AGL21_-2021TOPO	5’-CACCCACAGCAAAGATAAACACACACAATTAC-3’	AGL21 promoter-GFP-NLS
AGL21_-1R	5’-CAATTTTATCCTCTAATTGAATCTCCTCTG-3’	AGL21 promoter-GFP-NLS
ANR1_-2032TOPO	5’-CACCGAGCGTGAGGGAGGTATGTTAG-3’	ANR1 promoter-GFP-NLS
ANR1_-1R	5’-CTCTCTCCCCAAAAGACTAACAC-3’	ANR1 promoter-GFP-NLS
SG2	5’-AAGCAACGTATGACATGAAAGATGC-3’	Gene specific primer of *ANR1*
SG10	5’-GACATTCATGCGCATTTCC-3’	Gene specific primer of *ANR1*
dSpm1	5’-CTTATTTCAGTAAGAGTGTGGGGTTTTGG-3’	*dSpm* specific primer

SG3	5'-GAACCCCGCATCAGAAGTCAAG-3'	Gene specific primer of *AGL21*
SG4	5'-GTGTTGTGTGTTACAGTTTTGGCAG-3'	Gene specific primer of *AGL21*
SG6	5'-GAATCCGGAAAAGCAATCGTTC-3'	Gene specific primer of *AGL21*
dSpm8	5'-GTTTTGGCCGACACTCCTTACC-3'	*dSpm* specific primer
